# From health protection to labor incentive: health and economic impacts of Medical Financial Assistance in rural China

**DOI:** 10.3389/fpubh.2026.1833983

**Published:** 2026-05-18

**Authors:** Yarong Wang, Weikun Yang, Shi Yin

**Affiliations:** 1 College of Economics and Management, Hebei Agricultural University, Baoding, China; 2 Hebei Provincial Key Laboratory of Science and Technology Finance, Hebei Finance University, Baoding, China; 3 College of Humanities and Social Sciences, Hebei Agricultural University, Baoding, China

**Keywords:** health protection, low-income rural households, Medical Financial Assistance, non-farm labor participation rate, non-farm labor time

## Abstract

Medical Financial Assistance (MFA), a key safety net for low-income groups in China, may exert incentive or welfare dependency effects on non-farm labor supply, an issue critical to assessing the effectiveness of medical security policies. Using two-wave household panel data, this study employs two-way fixed effects models to evaluate MFA’s impact on the non-farm labor supply (participation rate and working time) of low-income rural households, distinguishing between the effects of eligibility attainment and increased assistance amounts. The results show that: (1) MFA eligibility significantly enhances non-farm labor participation (by 2.0 percentage points) and working time (by 4.87 days), confirming an incentive effect with marked localization bias (no significant impact on non-local employment); (2) increased assistance amounts have a negligible marginal incentive effect, reflecting a threshold characteristic where eligibility is the critical trigger; (3) policy effects are heterogeneous: Eligibility boosts participation rates most among older adult households (3.6 percentage points), households with major diseases (9.5 percentage points), and those without employment assistance (2.5 percentage points); (4) for working time, prime-age households (6.49 days), households with major diseases (36.00 days), and those with employment assistance (4.55 days) benefit the most. A limitation is MFA’s insignificant effect on non-local non-farm employment and weak marginal effects of increased assistance. Based on microdata from deep poverty areas, this study reveals MFA’s labor supply incentive effects, localization bias, and threshold characteristics, providing Chinese empirical evidence for understanding the labor market consequences of medical security systems in developing countries and informing health and social care policy design.

## 1 Introduction

Health is a core component of human capital that directly affects labor productivity and market labor supply capacity (1). Health shocks not only impair a patient’s own labor capacity but also create rigid household caregiving demands, thereby crowding out the labor time of other family members (2, 3). The resulting direct medical expenditures and indirect income losses may push households into poverty due to illness. It is estimated that nearly 100 million people globally fall into poverty each year because of catastrophic health expenditures (4, 5). Among these, the health depreciation and decline in labor supply capacity of the working-age population constitute a key transmission channel for the persistent deterioration of household poverty (6). Effectively mitigating the suppression of household labor supply caused by health shocks through institutional design has become a core concern for countries seeking to improve their social security systems (7, 8). This transition coincides with a critical juncture in China’s healthcare system as it shifts from “broad coverage” to “high-quality development”—the fiscal pressure on basic medical insurance funds continues to escalate, and as a fiscal backstop tool, the sustainability and incentive compatibility of Medical Financial Assistance (MFA) are facing unprecedented structural tests.

As the world’s largest developing country, China eliminated absolute poverty in 2020, but consolidating this achievement still faces severe challenges. Dynamic monitoring data on poverty prevention show that households at risk of poverty due to illness account for a persistently high proportion among monitored subjects. Data indicate that since 2021, among the cumulatively monitored “three types of households” (households that have just escaped poverty, households on the poverty margin, and households experiencing sudden hardships), nearly 1.08 million households faced the risk of poverty due to illness, representing 48.13% of all monitored households (9). This reality suggests that in the post-poverty alleviation era, health risk shocks have become a key factor affecting the livelihood stability of formerly poor populations (10). Activating the endogenous development momentum of low-income rural households focuses on enhancing their labor supply capacity, particularly for non-farm labor. Non-farm employment represents a primary pathway for rural households to transcend resource constraints, achieve income diversification, and integrate into the modern economic system. Agricultural labor, by contrast, is often constrained by limited land endowments, seasonal production cycles, and exposure to natural risks, which together restrict its capacity for sustained income growth. For low-income rural households, non-farm employment offers higher marginal returns and stronger poverty reduction effects, making it a more promising channel for activating endogenous development momentum. Therefore, understanding how public policies influence non-farm labor supply is crucial for designing effective poverty prevention strategies. In this article, non-farm labor in rural areas is defined as any income-generating activity outside the agricultural sector, including wage employment, self-employment, and temporary or public welfare positions such as epidemic prevention duties, rural sanitation work, and forest ranger roles. Unlike urban non-farm employment, which typically features formal contracts, stable working hours, and social insurance coverage, rural non-farm labor is often characterized by informality, seasonality, and a higher prevalence of part-time or flexible arrangements. This distinction is essential for accurately measuring labor supply responses and for understanding how social policies affect the livelihood strategies of low-income rural households.

Within China’s multi-tiered medical security system, Medical Financial Assistance (MFA) is positioned as a safety-net institutional arrangement targeting low-income groups. Unlike universal basic medical insurance, MFA, as an ex-post cash transfer program, aims to compensate for medical expenses for eligible households and directly offset the catastrophic health expenditure risks they face. In 2021, the General Office of the State Council issued the Opinions on Improving the Medical Insurance and Assistance System for Major and Catastrophic Illnesses, marking a strategic transformation of the MFA policy from extraordinary poverty alleviation to routine poverty prevention. As funding continues to increase and coverage expands, this institutional arrangement may exert more profound effects on the labor supply decisions of recipient households, effects that are distinct from those of universal health insurance.

Theoretically, MFA may affect labor supply through two pathways. First, through cost compensation, it reduces the burden of household medical expenditures, freeing up household resources originally allocated for medical expenses. Second, by ensuring timely treatment, it helps restore human capital and, by alleviating household caregiving burdens, releases bound labor time (11). Unlike the potential welfare dependency associated with the Dibao program (Minimum Living Standard Guarantee System), existing studies have found that MFA does not significantly suppress labor supply (12), providing preliminary evidence for exploring its positive effects.

However, existing research on the impact of medical security on labor supply mostly remains at the aggregate level, failing to distinguish between the two dimensions of labor participation decisions and labor time input, and paying limited attention to differences in employment location choices (13). The MFA policy encompasses two observable intervention dimensions: eligibility attainment and assistance amount. These two dimensions may have different mechanisms affecting rural household labor supply decisions. This distinction has not received adequate attention in current research. Furthermore, the manifestation of policy effects across heterogeneous dimensions such as family life cycle and disease type remains unclear, making it difficult to provide a scientific basis for targeted policy implementation (14).

Based on this, using two-wave household panel data from 2021 to 2022 in County L, a nationally designated deep poverty-stricken county in the Yanshan-Taihang Mountain region of Hebei Province, this study focuses on the impact of the MFA policy on the non-farm labor supply of low-income rural households, seeking to answer the following questions: Can MFA significantly increase the non-farm labor participation rate and labor time of low-income rural households? Does this promotional effect differ between local employment and non-local employment? Is the policy effect primarily derived from the threshold effect of eligibility attainment or the intensity effect of increased assistance amounts? Does the policy effect exhibit significant heterogeneity across the three dimensions of family life cycle, disease type, and employment assistance status?

The marginal contributions of this study are as follows. First, it systematically elucidates the internal logic through which MFA affects non-farm labor supply from the perspectives of health restoration, time release, and constraints on employment location choice. Second, it systematically examines the impact of MFA on non-farm labor supply from both the extensive and intensive margins, further distinguishing policy effects between local and non-local employment, revealing the locational structure of MFA policy effects. Third, it distinguishes between the eligibility effect and the intensity effect of MFA, revealing the threshold characteristics of policy incentives by comparing the differential impacts of eligibility attainment and increased assistance amounts on non-farm labor supply. Fourth, it analyzes the heterogeneity of policy effects across family life cycle, disease type, and employment assistance status, providing empirical evidence for accurately identifying the boundaries of policy effects.

The remainder of this article is organized as follows. Section 2 reviews the relevant literature on medical security and labor supply, health shocks and household care, and labor mobility and employment location choice. Section 3 constructs the theoretical analytical framework and proposes research hypotheses. Section 4 describes the data sources, variable definitions, and model specifications. Section 5 reports the sample characteristics and empirical results of the baseline regression, robustness checks, and heterogeneity analysis, and discusses these findings. Section 6 summarizes the research conclusions and elaborates on policy implications.

## 2 Literature review

As a safety-net institutional arrangement within the multi-tiered medical security system, the Medical Financial Assistance (MFA) policy can be examined from two analytical dimensions: burden reduction and empowerment. This study focuses on the impact of MFA on the non-farm labor supply of low-income rural households. It reviews the literature around three core themes: the dual effects of medical security on labor supply, the suppression mechanism of health shocks on household time allocation, and labor mobility and employment location choice. By clarifying the contributions and limitations of existing research, this section establishes the foundation for the theoretical framework and empirical analysis of this study.

### 2.1 Medical security and labor supply: health empowerment or income suppression

The impact of medical security on labor supply is a classic topic in labor economics and health economics, with existing research presenting a theoretical debate on dual effects. On the one hand, medical security may promote labor supply through a health empowerment channel. The core logic is that medical security, by lowering the economic threshold for healthcare access, improves enrollees’ healthcare accessibility and actual utilization, thereby enhancing health status and rebuilding health human capital (1). Health improvement can directly enhance workers’ productivity, effective working hours, and employment stability (15), ultimately translating into higher labor participation rates and longer working hours.

A substantial body of empirical research provides supporting evidence for this mechanism. Studies on Medicaid in the United States find that public health insurance can promote labor participation by improving the physical and mental health status of low-income populations, particularly aiding individuals who left the labor market due to health issues in re-entering employment (16). Using quasi-experiments from Medicaid introductions, research further confirms that eligible single mothers did not experience significant declines in labor supply but instead showed positive point estimates, suggesting that health gains may offset potential labor disincentive effects (17). Other studies reveal positive effects of Medicaid expansion on labor supply while also noting potential employment lock effects (18). Research based on ACA Medicaid expansion finds that in the 15 months following expansion, employment, job transitions, and full-time or part-time status among low-income adults did not change significantly (19), providing important insights for understanding the threshold effect of attaining medical insurance eligibility.

In developing country research, analysis based on China Nutrition and Health Survey data finds that the expansion of the New Cooperative Medical Scheme positively affects both agricultural labor time and non-farm labor participation, with effects more pronounced among males, individuals over 50, and low-income households, confirming the important role of health improvement in promoting rural residents’ labor productivity (13). Using data from the Oregon Health Insurance Experiment, research further confirms the positive impact of Medicaid expansion on labor supply (20). Utilizing the gradual reform of urban–rural health insurance integration, research confirms that insurance integration significantly reduces rural residents’ poverty vulnerability by enhancing labor supply and health checkups (10), providing direct evidence that labor supply serves as a mediating channel through which medical insurance policy affects household welfare. Research on China’s Urban Resident Basic Medical Insurance reveals complex impacts on the labor market, finding that while URBMI did not significantly change average labor participation rates, it increased employment mobility, manifested as reductions in long-term employment and increases in fixed-term contracts and self-employment (21), highlighting the potential role of medical insurance in mitigating employment lock effects. Recent research further indicates that urban–rural basic medical insurance integration significantly promotes the probability and quality of non-farm employment among rural laborers through three channels: alleviating liquidity constraints, enhancing health human capital, and broadening employment location choices (22).

On the other hand, medical security may also generate income effects or employment lock effects that suppress labor supply. According to the neoclassical labor-leisure model, any increase in non-labor income may produce a pure income effect, leading individuals pursuing utility maximization to reduce labor supply to purchase more leisure (3). Studies find that public insurance eligibility is associated with declines in full-time employment and increases in part-time employment and non-work probability among low-income childless adults, with this association more pronounced among those in poorer health and aged 50 to 64 (23). Research based on developed countries finds that medical insurance expansion, particularly for groups nearing retirement, may provide a safety net for exiting the labor market, thereby encouraging early retirement (24). Studies on the ACA confirm that premium subsidies and Medicaid expansion, respectively, reduced labor participation rates among those aged 55 to 64, indicating that expanded insurance coverage may incentivize early retirement among older workers (25). Additionally, employment-linked health insurance may create employment lock effects, where workers hesitate to change jobs or start businesses to avoid losing existing medical coverage (26). Research based on CHARLS data finds that the New Cooperative Medical Scheme actually reduced enrollees’ non-farm labor participation rates and working hours (27), lending support to the predominance of income effects. Studies on low-income populations in Vietnam also find suppressive effects of medical security on labor supply (28).

Why do studies on similar policies yield seemingly contradictory conclusions? Systematic differences in research subjects, methodologies, and institutional contexts are key to unraveling this puzzle. First, the life cycle stage of research subjects is a core dimension leading to effect differentiation. For older adults nearing retirement, the income effect from medical security often dominates; for prime-age workers, the substitution effect triggered by health improvements through medical security may be stronger (29). Second, the type and design characteristics of coverage also lead to complex changes in labor supply behavior. Research finds that migrant workers with access to medical insurance in destination areas have shorter labor supply hours but higher labor supply quality, revealing a quality-quantity trade-off through which insurance affects labor supply (30). Research on Mexico’s Seguro Popular provides another perspective, finding that the insurance expansion reduced the probability of female informal workers exiting the labor market, with further analysis indicating this effect operates mainly by reducing health shocks to family members, enabling caregivers to continue working (31), thereby providing a new theoretical channel for understanding how medical security affects labor supply through intra-household spillover effects.

Notably, direct research on China’s rural MFA policy has not provided strong evidence for the aforementioned negative effects. Based on China Household Finance Survey data, research finds that unlike the potential welfare dependency associated with the Dibao program (Minimum Living Standard Guarantee System), MFA does not exhibit significant suppressive effects on labor supply (12). This suggests that for low-income households facing extremely tight budget constraints and urgent health needs, the marginal utility of the empowerment effect—alleviating rigid expenditure shocks and releasing productive resources—likely far outweighs any negative income effect stemming from a one-time transfer payment. This judgment aligns with theoretical analysis: MFA releases household labor by safeguarding health, thereby generating positive economic behavioral responses (32).

### 2.2 Health shocks, household care, and labor supply

Health shocks are core risks that contribute to the vicious cycle of poverty and illness among vulnerable households. Their suppressive mechanism on labor supply can be understood from two dimensions: health capital inhibition and time endowment inhibition.

From the health capital dimension, Grossman’s health capital theory conceptualizes health as a core productive capital that depreciates but can be maintained through investment (1). Health shocks directly erode this capital stock, undermining workers’ productivity (33). A substantial body of empirical research confirms this mechanism. Based on data from the China Health and Nutrition Survey, research finds that health has significant positive effects on both labor participation and working hours, with this effect exhibiting clear life cycle characteristics (34). Using multi-country European data, research confirms that health shocks significantly increase individuals’ probability of exiting employment, with this effect varying across countries and being closely related to social security institutional arrangements (35). Studies on 16 European countries find that acute health shocks have significant negative effects on older workers’ labor supply (36). Further research reveals the mechanisms through which health affects labor supply among groups nearing retirement (37). Domestic scholars also confirm the negative impact of health shocks on labor supply among rural middle-aged and older adult individuals (38).

From the time endowment inhibition dimension, integrating Becker’s (2) household production theory and Gronau’s (3) three-way time allocation model, illness not only reduces the patient’s productivity but also inevitably crowds out other family members’ caregiving time, effectively reducing the household’s total time endowment available for market activities. Research confirms that informal care has significant negative effects on caregivers’ wages and employment (39). Using UK longitudinal data, research finds no significant added worker effect following health shocks but rather a significant informal care effect, where spouses substitute care time for leisure time (40), providing a new perspective for understanding household time allocation responses to health shocks. Research on China finds that parental health shocks lead to persistent declines in female employment for at least 6 years, with insignificant changes in male employment, revealing the long-term nature and gender differences in health shock effects on household labor supply (41). Using data from Indian agricultural households, research finds that short-term illness shocks reduce individuals’ monthly wage income while triggering compensatory labor supply from spouses, although these compensation effects only partially offset income losses (42). Using German Socio-Economic Panel data, research finds that affected individuals suffer persistent labor income losses following health shocks, with labor participation declining and females significantly increasing household production time (43), further confirming labor reallocation effects triggered by health shocks within households.

In the Chinese context, research finds that family caregiving responsibilities significantly suppress caregivers’ labor market participation, particularly among females (44). Other studies indicate that older adult parent care reduces adult children’s labor supply (45). Based on CFPS data, research shows that children’s health problems significantly reduce parents’ labor supply (46). Studies reveal significant gender differences in how family care affects labor participation (47). Recent research finds that spousal disability has significant negative effects on female employment lasting over 8 years, with negligible effects on males. This effect is more pronounced among groups with lower education levels, agricultural household registration, and stronger Confucian cultural influence (48), highlighting the important role of institutional environment and cultural norms in moderating the effects of health shocks.

The aforementioned suppressive effects of health shocks are not uniform, with their severity closely related to household labor composition. Research confirms that the degree to which informal care effects from health shocks suppress household labor supply depends significantly on intra-household labor composition and role division (40). Research on China’s New Cooperative Medical Scheme confirms that the impact of medical security policies varies significantly across groups with different health statuses (49). Studies indicate that disease severity is a key factor leading to differences in medical needs, economic shocks, and household coping strategies (50). Further research finds that paid family leave policies influence labor supply decisions following spousal health shocks, revealing the moderating role of institutional arrangements in balancing family care and work (51), providing important insights for understanding how external policies intervene in intra-household time allocation.

### 2.3 Labor mobility, migration costs, and employment location choice

Non-farm employment is an important pathway for rural households to overcome resource constraints and achieve income diversification. However, labor mobility is not costless, and location choice is constrained by multiple factors. According to internal labor market theory proposed by Doeringer and Piore (52), workers entering non-local employment markets face not only explicit migration costs but also entry barriers created by factors such as information asymmetry and social network deficits.

Recent research on employment location choice increasingly focuses on structural differences between local and non-local employment and their determinants. From the perspective of county-level industrial specialization in China, studies find that higher county industrial specialization increases rural laborers’ propensity to choose local non-farm employment over out-migration, enabling them to secure more stable and formal non-farm work through their own efforts rather than relying on social networks (53). This provides direct evidence for understanding the mechanisms underlying localization bias. Analyzing seasonal migration subsidy experiments in Bangladesh, researchers find that the welfare gains from migration subsidies primarily result from providing insurance to vulnerable rural households rather than correcting spatial misallocation (54), offering theoretical support from a welfare economics perspective for the social protection function of local employment support. Other research highlights potential negative effects of migration on human capital accumulation. When the returns to education from rural–urban migration are lower than those in rural areas, relaxing migration restrictions diminishes human capital accumulation (55), providing a new explanatory perspective for understanding why some laborers choose to remain locally rather than migrate.

In the Chinese context, research on labor mobility and employment location choice has yielded significant findings. Additional studies reveal the complex effects of insurance coverage and migration intentions on migrant workers’ labor supply (30). Based on large-scale national household survey data, research shows that a 10% increase in non-farm employment raises the probability of farmland abandonment, illustrating the impact of labor outflow on agricultural production (56), and indirectly indicating the agricultural protection effect of local labor allocation. Research indicates that poverty alleviation policies with Chinese characteristics effectively promote labor supply among local non-migrant households (57), offering insights into MFA’s impact on local employment. Studies explore the influence of medical insurance on labor migration decisions, finding that medical insurance is a crucial factor affecting rural labor migration (58). Further research examines the effects of urban–rural health insurance integration policies on the labor supply among flexible workers (59). Recent studies from the perspective of employment location choice investigate the impact of non-farm employment on fertilizer reduction, finding that local and non-local employment have opposite effects on fertilizer use, with local employment leading to negative effects and non-local employment resulting in positive effects (60). This reveals that employment location choice not only affects household labor allocation but also profoundly impacts agricultural production through land-use behavior.

During the 2021–2022 COVID-19 pandemic, labor mobility patterns changed significantly. International studies indicate that the pandemic caused sharp shocks to low-wage service sector employment (61), while disruptions to childcare and education led to significant reductions in labor supply among parents, particularly mothers (62), imposing disproportionate economic costs on women, low-wage workers, and young people (63, 64). This unique period provides an important macro context for examining MFA’s employment stabilization function under systemic shocks.

Additionally, some studies have begun to focus on the complex interrelationships among health, migration, and employment. Research finds that individuals with better health are more likely to migrate, with social networks playing a mediating role (65), highlighting the importance of health selection in migration decisions. Other research indicates that cross-province migration actually worsens migrants’ health status (66), providing evidence of the health costs associated with migration. Studies explore the impact of medical insurance on migrant workers’ healthcare utilization, finding that insurance participation increases migrants’ likelihood of seeking medical care, although the New Cooperative Medical Scheme fails to function effectively (67), exposing the constraints imposed by institutional segmentation on migrant populations’ health protection. Recent research further finds that while migrant populations have similar public insurance participation rates to urban residents, their commercial insurance participation rates are significantly lower (68), illustrating institutional barriers that restrict migrant populations’ access to medical security.

### 2.4 Summary

Through a systematic review of the aforementioned literature, it is evident that existing research provides a solid theoretical foundation for this study. However, several areas warrant further exploration.

First, the understanding of MFA policy objectives requires expansion. Existing research primarily focuses on MFA’s burden reduction function, namely reducing out-of-pocket medical expenses and mitigating catastrophic health expenditures. While this is undoubtedly the policy’s core objective, policy evaluation should not be confined to examining cost compensation alone but should also consider its impact on household economic behavior. Although the empowerment effect of medical security has attracted scholarly attention, direct research systematically examining MFA’s impact on non-farm labor supply remains relatively limited. Unlike universal medical insurance, MFA, as a targeted safety-net institutional arrangement for low-income groups, possesses distinctive features, including ex-post compensation mechanisms, precise targeting characteristics, and direct relaxation of budget constraints. These features may uniquely influence recipient households’ labor supply decisions. This research gap urgently needs to be addressed.

Second, research perspectives require integration, and theoretical frameworks need to be constructed. Existing literature largely examines the impact of medical security on labor supply, the crowding-out of household time by health shocks, and the location choice of labor mobility in isolation. It fails to systematically situate MFA, a specific safety-net policy, within the complete causal chain of health shocks, household behavioral responses, and employment location choice. A systematic analytical framework integrating health human capital theory, household time allocation theory, and internal labor market theory is lacking to understand how MFA affects intra-household labor resource allocation and employment location choice as core micro-behavioral mechanisms. Accordingly, this study constructs a binary theoretical analytical framework of empowerment and orientation, incorporating MFA’s health restoration effects, time release effects, and localization constraints on employment location choice into a unified analytical system. This aims to provide systematic theoretical explanations for understanding MFA’s mechanisms affecting non-farm labor supply.

Third, the multidimensional measurement of policy effects and attention to locational heterogeneity are insufficient. MFA’s impact on non-farm labor supply can be examined from two dimensions: the extensive margin, concerning non-farm labor participation decisions, and the intensive margin, concerning non-farm labor time allocation. Existing research rarely examines MFA’s policy effects from both dimensions simultaneously in a systematic manner. More importantly, location choice in labor mobility is a key factor for understanding rural households’ employment decisions. Local and non-local employment differ fundamentally in terms of migration costs, information acquisition, and compatibility with family care. This locational dimension has yet to be fully incorporated into analytical frameworks examining MFA’s policy effects. How MFA specifically affects rural households’ employment choices between local and non-local options remains an important question requiring urgent investigation.

Fourth, the heterogeneity in policy effects demands deeper exploration. MFA’s impact on non-farm labor supply may exhibit systematic differences depending on households’ life cycle stages, types of illnesses experienced, and employment assistance receipt status. Heterogeneity in the economic consequences of health shocks across different groups has been widely confirmed, with their suppressive effects on caregivers’ labor supply also varying by intra-household labor composition and role division. This implies that when households with different age structures and labor endowments encounter health shocks, the suppressive effects on their labor supply differ, potentially moderating MFA’s labor supply promotion effects. However, these heterogeneity dimensions have yet to be systematically incorporated into analytical frameworks examining MFA’s policy effects. MFA’s differential impacts on non-farm labor supply across different household types remain an important question requiring further exploration.

Finally, the distinction between MFA’s eligibility effect and intensity effect requires clarification. The MFA policy encompasses two observable intervention dimensions: whether households receive eligibility and the amount of assistance they obtain. These two dimensions may have different mechanisms influencing household labor supply decisions. Eligibility attainment itself may generate threshold effects by stabilizing household expectations and triggering health investment behaviors, while increased assistance amounts may produce marginal incentive effects by alleviating medical expenditure burdens. Existing research inadequately distinguishes these two dimensions and lacks a systematic examination of their differential impacts.

Based on this, this study focuses on the impact of government-led MFA on low-income rural households’ non-farm labor supply. It systematically examines the effects of MFA eligibility attainment and assistance amounts on non-farm labor participation rates and labor time from both extensive and intensive margins, and further investigates differences in policy effects between local and non-local employment. Simultaneously, it deeply analyzes heterogeneity in policy effects across three dimensions, including family life cycle, disease type, and employment assistance status, aiming to address gaps in existing research.

## 3 Theoretical analysis and research hypotheses

### 3.1 Theoretical analysis

As an ex-post cash transfer program administered by the government, Medical Financial Assistance serves a core function of directly offsetting household out-of-pocket medical expenses through its reimbursement mechanism, thereby playing a financial safety-net role (69). However, building upon Grossman’s health demand theory (1) and Becker’s household production theory (2), the policy impact of MFA extends far beyond financial infusion through medical expense compensation. It may also empower recipient households by restoring health human capital and freeing up caregiving time.

Low-income rural households experiencing health shocks face three main constraints on their labor supply. The first is a financial constraint. Catastrophic medical expenditures directly deplete household savings and liquid assets. By exacerbating uncertainty about future expenses, they force households to increase precautionary savings, thereby crowding out liquid resources available for production and consumption and leading to a significant tightening of household budget constraints (70). The second is a health capital constraint. According to Grossman’s health capital theory, severe illness leads to accelerated depreciation of health human capital, directly undermining worker productivity and market labor supply capacity (33). The third is a time endowment constraint. Integrating Becker’s household production theory, severe health shocks not only erode the patient’s own productive capacity but also create rigid needs for treatment and care, limiting the time that other family members could otherwise devote to market labor or household production. This consumes the household’s total effective time endowment. Research shows that family caregiving responsibilities significantly suppress caregivers’ labor market participation (44).

The empowerment effect of the MFA policy operates through two pathways. The first is the health capital restoration pathway. MFA, by lowering the effective price of medical services, improves healthcare accessibility and utilization efficiency (32), thereby promoting health investment and restoring damaged health human capital (71). Health improvement can directly enhance worker productivity and market labor supply capacity (33). The second is the time endowment release pathway. Effective treatment and recovery of patients directly reduce the caregiving time required from family members. According to Gronau’s three-way time allocation model (3), this released time endowment will be reallocated. Given that the marginal returns to non-farm labor typically exceed those of agricultural labor, this time is likely to be preferentially allocated to non-farm employment (72, 73).

The labor supply potential thus stimulated faces multiple structural constraints when translated into actual employment, resulting in a systematic localization bias. For low-income rural households experiencing health shocks, non-local non-farm employment presents higher entry barriers. Internal labor market theory proposed by Doeringer and Piore indicates that workers entering non-local employment markets must bear not only explicit migration costs but also overcome entry barriers created by information asymmetry and social network deficits (52). Subsequent elaboration of this theory further reveals the intrinsic relationship between labor market segmentation and location choice (74). In the research context of this study, these barriers manifest in three aspects: health thresholds, migration costs, and family responsibilities. Non-local employment typically requires continuous high-intensity labor input, which recovering workers may find difficult to sustain (71). Explicit costs such as transportation and accommodation, along with psychological costs associated with family separation, create migration cost barriers. Ill family members may require intermittent follow-up visits or daily care, prompting healthy household members to consider caregiving responsibilities when choosing employment locations (72). Constrained by these multiple factors, released labor tends to prefer local non-farm employment.

Crucially, the magnitude of these two recovery pathways is not uniform across all households. It varies systematically with three dimensions of household heterogeneity that determine the baseline constraints MFA aims to alleviate: family life cycle, health shock severity, and employment assistance status.

First, according to family life cycle theory, households at different stages exhibit significant differences in labor endowment, caregiving responsibilities, and economic objectives (75). Second, drawing on health demand theory, the severity of the health shock directly affects the extent of health capital depletion. Households experiencing major disease shocks have the greatest potential for health improvement; therefore, both recovery pathways should be significantly stronger for them than for those with minor or chronic conditions (1). Third, labor market segmentation theory implies that employment assistance status reflects a household’s connection to external markets. Households without such support face higher entry barriers; MFA thus first helps them cross the participation threshold (extensive margin). Households with employment assistance already have some market connection, so MFA’s effect is more likely to manifest in extending working time (intensive margin) (52).

**Figure 1:**
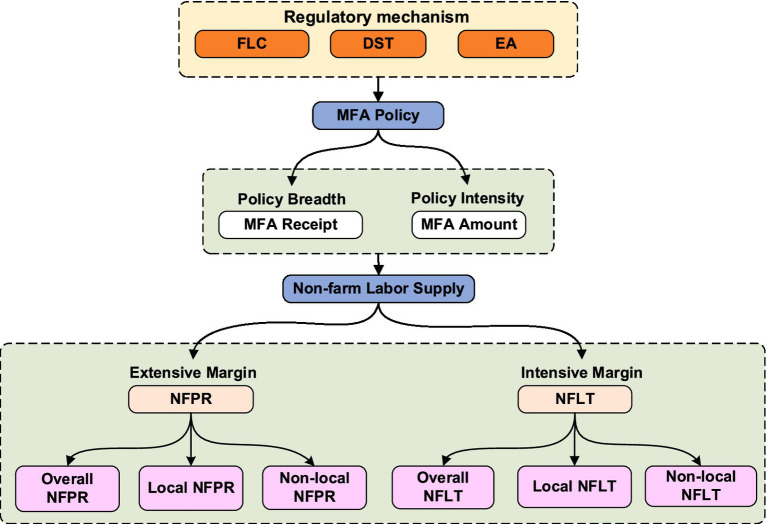
Analytical framework. NFPR denotes non-farm labor participation rate; NFLT denotes non-farm labor time (days); FLC denotes family life cycle; DST denotes disease shock type; EA denotes employment assistance; MFA denotes Medical Financial Assistance.

Based on the above analysis, this study constructs a theoretical framework to explain how MFA affects rural households’ non-farm labor supply, as shown in Figure 1. The core logic of this framework is that MFA, through cost compensation, alleviates household budget constraints, thereby restoring health capital and freeing up household labor time previously occupied by caregiving demands, ultimately stimulating non-farm labor supply potential. Under the multiple constraints of health thresholds, migration costs, and family responsibilities, released labor tends to prefer local non-farm employment. This framework extends the policy effect of MFA from a mere financial safety net to labor supply incentives. Drawing on Grossman’s health demand theory and Becker’s household production theory, it clarifies two theoretical pathways through which MFA affects labor supply. Integrating Doeringer and Piore’s labor market segmentation theory, it explains the formation mechanism of locational differentiation in policy effects. Importantly, the framework incorporates the three dimensions of household heterogeneity discussed above as moderating variables, which delineate the boundary conditions under which the two recovery pathways operate.

### 3.2 Model deduction and research hypotheses

To formalize the above logic, consider the utility maximization problem of a low-income rural household experiencing a health shock. Assume household utility derives from consumption *C* and leisure *L*, with U=U(C,L) satisfying *U_C_* > 0 and *U_L_* > 0. Low-income rural households face the following constraints. When a household faces catastrophic medical expenses, its utility maximization problem is actually subject to *C* ≥ max(0, M - S) (where M is medical expenditure and S is savings). At this point, obtaining MFA eligibility directly relaxes this constraint, which is the micro-foundation of the “threshold effect.”

#### 3.2.1 Medical assistance and health capital restoration

According to Grossman’s (1) health demand model, health capital stock *H_it_* is endogenously determined by medical service utilization. Let *D_it_* indicate whether household *i* receives MFA in year *t*, and let *A_it_* represent the annual MFA amount received by the household. The health production function is specified as


$$C_{1}+S=Y_{1}-E(X)$$ (1)

In Equation 1, MFA affects health capital through two channels: Eligibility *D_it_* triggers health investment behavior, while assistance amount *A_it_* affects the intensity of health investment, satisfying *∂H_it_/∂D_it_* > 0 and *∂H_it_/∂A_it_* > 0. Health capital stock directly affects labor efficiency *e_it_* = *e*(*H_it_*), with *e’*(*H_it_*) > 0. The effective wage rate when household members participate in non-farm labor is *ωe_it_* = *ω·e(H_it_)*, where *ω* is the market benchmark wage rate.

This health production mechanism indicates that MFA can enhance worker productivity and market labor supply capacity by restoring health human capital. This mechanism provides a theoretical basis for examining the overall effect of the policy on non-farm labor supply. Accordingly, hypotheses H1a and H2a are proposed. Given the dynamic cumulative nature of health status, this study employs entropy balancing matching combined with difference-in-differences in the empirical study for robustness testing (see Section 5.3.3) to mitigate potential selection bias and reverse causality.

*Hypothesis H1a:* Obtaining MFA eligibility significantly increases the non-farm labor participation rate of low-income rural households.

*Hypothesis H2a:* Obtaining MFA eligibility significantly increases the non-farm labor time of low-income rural households.

#### 3.2.2 Release effect of health improvement on household time allocation

According to Gronau’s (3) three-way time allocation model, the household’s total time endowment *T* is allocated among non-farm labor *N*, household production *H*, and leisure *L*, as shown in Equation 2:


$$T_{\mathrm{it}}=T_{\mathrm{it}}^{n}+T_{\mathrm{it}}^{h}+L_{\mathrm{it}}\;$$ (2)

Household production time includes caregiving time $T_{\mathrm{it}}^{c}\;$ required by the patient, which is a decreasing function of health level $T_{\mathrm{it}}^{c}=T_{\mathrm{it}}^{c}\;(H_{\mathrm{it}}\;)$ with ${\left(T_{\mathrm{it}}^{c}\;\right)}^{\prime }(H_{\mathrm{it}}\;)<0$ . MFA, by improving health, reduces caregiving time, yielding $\partial T_{\mathrm{it}}^{c}/\partial D_{\mathrm{it}}<0$ and $\partial T_{\mathrm{it}}^{c}/\partial A_{\mathrm{it}}<0$ . The released time $\Delta T_{\mathrm{it}}^{m}=-\Delta T_{\mathrm{it}}^{c}>0$ can be reallocated to market labor. Integrating the health restoration effect (increasing $\phi (H_{\mathrm{it}})$ ) and the time release effect (increasing $\Delta T_{\mathrm{it}}^{m}\;$ ), the change in optimal household non-farm labor supply is


$$\Delta T_{\mathrm{it}}^{n*}=f(\Delta \phi (H_{\mathrm{it}}\;),\Delta T_{\mathrm{it}}^{m}\;)>0$$ (3)

Here, non-farm labor participation status *P_it_* is defined such that *P_it_* = 1 if $\;T_{\mathrm{it}}^{n^{*}}>0$ , and *P_it_* = 0 otherwise. Equation 3 indicates that MFA, by improving health and reducing caregiving time, releases household labor and thereby increases non-farm labor supply. This mechanism further supports the theoretical expectations of hypotheses H1a and H2a.

#### 3.2.3 Employment location choice in non-farm labor supply

Decompose non-farm labor time into local $T_{\mathrm{it}}^{l}\;$ and non-local $T_{\mathrm{it}}^{m}$ components, with $T_{\mathrm{it}}^{n}=T_{\mathrm{it}}^{l}+T_{\mathrm{it}}^{m}$ . According to Doeringer and Piore’s internal labor market theory (52), non-local employment entails migration costs *C_m_* and utility discounting from family separation, incorporated into the household utility function as *U(C, L, D)*, where $D(T_{\mathrm{it}}^{m}\;)$ captures utility loss from family separation. The optimal allocation condition for household labor between local and non-local employment is


$$\omega_{l}\cdot \phi (H_{\mathrm{it}}\;)=\omega_{m}\cdot \phi (H_{\mathrm{it}}\;)-c_{m}-D^{\prime }(T_{\mathrm{it}}^{m}\;)$$ (4)

In Equation 4, *ω_l_* and *ω_m_* represent local and non-local wage rates respectively, typically with *ω_m_* > *ω_l_*. When migration costs and utility losses are sufficiently high such that $\Delta \phi (H_{\mathrm{it}}\;)<c_{m}+D^{\prime }(T_{\mathrm{it}}^{m}\;)$ , rural households will allocate all newly released labor supply to the local market, yielding $\Delta T_{\mathrm{it}}^{l}=\Delta T_{\mathrm{it}}^{n^{*}}$ and $\Delta T_{\mathrm{it}}^{m}=0$ . Define local participation status *P_l_* and non-local participation status *P_m_*, where *P_l_* = 1 if $T_{\mathrm{it}}^{l}>0$ , and *P_m_* = 1 if $T_{\mathrm{it}}^{m}>0$ .

When migration costs and family responsibility constraints are sufficiently high, low-income rural households will prioritize allocating newly released labor to the local market. This mechanism provides a theoretical explanation for the localization characteristics of MFA policy effects. Accordingly, hypotheses H1b and H2b are proposed.

*Hypothesis H1b:* The promotional effect of obtaining MFA eligibility on the non-farm labor participation rate is primarily manifested in local non-farm labor participation, with limited effects on non-local non-farm labor participation.

*Hypothesis H2b:* The promotional effect of obtaining MFA eligibility on non-farm labor time is primarily manifested in increased local non-farm labor time, with limited effects on non-local non-farm labor time.

#### 3.2.4 Comparison between MFA eligibility effect and intensity effect

The above analysis focuses on the impact of MFA eligibility *Dit* on household labor supply. However, in practice, recipient households also experience variation in assistance variation in assistance amounts *A_it_*. From Equations 1, 3, it can be inferred that the restoration of health capital through MFA may exhibit threshold characteristics. Specifically, attaining eligibility is the critical factor triggering health investment behavior. Once the assistance amount reaches a level sufficient to cover basic medical needs, the marginal restorative effect of additional amounts on health capital may diminish to insignificance. This mechanism provides a theoretical basis for distinguishing between the extensive margin effect and the intensive margin effect of the policy. Accordingly, hypotheses H1c and H2c are proposed.

*Hypothesis H1c:* Increases in MFA amounts have no significant effect on the non-farm labor participation rate. The incentive effect of the policy primarily derives from attaining the eligibility threshold.

*Hypothesis H2c:* Increases in MFA amounts have no significant effect on non-farm labor time, further confirming the threshold characteristics of the policy effect.

#### 3.2.5 Heterogeneity effects hypotheses

Building on the theoretical framework established in Section 3.1, this subsection formally states the testable hypotheses regarding how MFA’s effects vary across household characteristics. The theoretical predictions for each moderator are summarized below, followed by the corresponding hypotheses.

##### 3.2.5.1 Heterogeneity in MFA eligibility effects

*Family life cycle*. According to the framework, older adult households are expected to show a larger increase in labor participation, while prime-age households are expected to show a larger increase in working time.

*Hypothesis H3a:* The promotional effect of obtaining MFA eligibility on the non-farm labor participation rate is most significant in older adult households with heavier caregiving burdens.

*Hypothesis H4a:* The promotional effect of obtaining MFA eligibility on non-farm labor time is most significant in prime-age households with more abundant labor endowments.

*Disease type*. The framework predicts that households affected by major diseases, due to their greater health capital loss, should exhibit stronger responses in both dimensions.

*Hypothesis H3b:* The promotional effect of obtaining MFA eligibility on the non-farm labor participation rate is most significant in households experiencing severe health shocks from major illnesses.

*Hypothesis H4b:* The promotional effect of obtaining MFA eligibility on non-farm labor time is most significant in households experiencing severe health shocks from major illnesses.

*Employment assistance status*. The framework predicts that households without employment support benefit more in terms of labor participation (extensive margin), while those with support benefit more in terms of working time (intensive margin).

*Hypothesis H3c:* The promotional effect of obtaining MFA eligibility on the non-farm labor participation rate is most significant in households without employment assistance.

*Hypothesis H4c:* The promotional effect of obtaining MFA eligibility on non-farm labor time is most significant in households with employment assistance that already possess external employment support.

##### 3.2.5.2 Heterogeneity in MFA intensity effects

From Equations 1, 3, it can also be inferred that among groups facing the strongest specific constraints, the marginal effect of assistance amounts may become apparent. For households lacking external employment support, their non-farm labor participation rate may be more sensitive to variations in assistance amounts. Similarly, for households with major illnesses, their non-farm labor time may also be more sensitive to variations in assistance amounts. Accordingly, hypotheses H3d and H4d are proposed.

*Hypothesis H3d:* In households without employment assistance that lack external employment support, increases in MFA amounts have a significant positive effect on the non-farm labor participation rate. In other households, no significant effect is observed.

*Hypothesis H4d:* In households with major illnesses experiencing the most severe health shocks, increases in MFA amounts have a significant positive effect on non-farm labor time. In other households, no significant effect is observed.

### 3.3 Mapping between theoretical parameters and observable variables

To connect the theoretical model with empirical analysis and clarify the testable implications of the theoretical deductions, this study establishes the following correspondence between theoretical parameters and observable variables. This mapping provides a clear theoretical foundation for subsequent variable selection, econometric model specification, and hypothesis testing, as detailed in Table 1.

**Table 1:** Correspondence between theoretical parameters and observable variables.

Theoretical parameter	Theoretical meaning	Observable variable	Hypotheses
$D_{\mathrm{it}}$	MFA eligibility attainment	Household MFA receipt status	H1a, H2a, H3a-c, H4a-c
$A_{\mathrm{it}}$	MFA support intensity	Annual household MFA amount (log)	H1c, H2c, H3d, H4d
$T_{\mathrm{it}}^{n}$	Non-farm labor time	Annual household non-farm labor days	H2a-c, H4a-d
$P_{\mathrm{it}}$	Non-farm labor participation decision	Household non-farm labor participation rate	H1a-c, H3a-d
$T_{\mathrm{it}}^{l}$	Local non-farm labor time	Non-farm labor days within the county	H2b, H4a-c
$T_{\mathrm{it}}^{m}$	Non-local non-farm labor time	Non-farm labor days outside the county	H2b, H4a-c
$P_{\mathrm{it}}^{l}$	Local non-farm labor participation	Local non-farm labor participation rate	H1b, H3a-c
$P_{\mathrm{it}}^{m}$	Non-local non-farm labor participation	Non-local non-farm labor participation rate	H1b, H3a-c
Family life cycle	Caregiving burden and labor endowment	Youth, middle-aged, older adults	H3a, H4a
Disease type	Health shock severity	Minor, chronic, major	H3b, H4b
Employment assistance	External market connection	With/without employment assistance	H3c, H4c

“H3a-c” indicates H3a, H3b, and H3c; “H2a-c” indicates H2a, H2b, and H2c; “H4a-d” indicates H4a, H4b, H4c, and H4d.

It should be noted that the non-farm labor participation rate *P_it_* in the theoretical model represents a binary participation status for an individual, while in empirical analysis it represents the aggregated proportion across multiple household members. When a household contains multiple laborers, the participation rate can be viewed as an extension of *P_it_* across household members. This treatment does not affect the testing of theoretical hypotheses.

## 4 Methodology

### 4.1 Data sources

The data for this study are derived from two rounds of household tracking surveys conducted by the research team from 2021 to 2022 in County L, a nationally designated deep poverty-stricken county in the Yanshan-Taihang Mountain region of Hebei Province. County L was selected as the research area because it serves as a typical deeply impoverished region and represents a key area for consolidating the achievements of poverty alleviation while preventing large-scale returns to poverty. Its policy practices provide important reference value for similar regions. To ensure the sample’s representativeness of the overall low-income rural household population in the county, the survey employed a stratified random sampling method with equal probability, covering 17 townships and 280 administrative villages in the county. Within sample villages, systematic sampling was conducted based on the complete rosters of low-income households provided by village committees. Structured questionnaires were used to systematically collect information on household demographics, health status, labor allocation, and participation in various policies, including Medical Financial Assistance. The initial sample contained 14,124 households across two waves. To precisely align with the research focus on non-farm labor supply behavior, this study conducted rigorous sample screening based on theoretical premises. First, households with no labor force members were excluded, which involved 583 households in 2021 and 629 households in 2022, as they lack the fundamental prerequisite for labor supply decisions. Second, groups such as those receiving Dibao (minimum living standard guarantee) for extremely poor individuals, whose survival depends entirely on government transfers, were excluded because their decision-making mechanisms differ systematically from those of ordinary low-income households. After the cleaning process and handling of missing values, a short panel dataset containing 13,515 farm households was finalized, with 13,102 households in 2021 and 13,091 households in 2022, demonstrating good representativeness.

### 4.2 Variable definitions

#### 4.2.1 Dependent variables

The dependent variable in this study is non-farm labor supply, defined as the labor decisions and time input behaviors of low-income rural households allocating their members’ labor to non-agricultural economic sectors to obtain remuneration, with the objective of maximizing household utility in the short term. It should be emphasized that non-farm labor in the rural context differs from its urban counterpart in several important ways. Urban non-farm employment is typically characterized by formal labor contracts, fixed working hours, social insurance coverage, and relatively stable job tenure. In contrast, rural non-farm labor is often informal, seasonal, and flexible, encompassing activities such as temporary wage work, self-employment in small businesses, and participation in public welfare programs. This definition establishes three research boundaries. Regarding the unit of analysis, it focuses on the aggregated performance of labor resource allocation at the household level. Regarding the industry scope, it explicitly excludes all types of labor within the agricultural sector, including self-employment and agricultural wage labor, to ensure the purity of non-farm attributes. Regarding the time dimension, it focuses on the quantity and time allocation of labor in the short term, without involving long-term human capital accumulation effects.

It should be noted that, considering the livelihood context during the COVID-19 pandemic from 2021 to 2022, this study extends the scope of non-farm labor to include various types of temporary and public welfare positions, such as epidemic prevention duties, rural sanitation workers, forest rangers, and flexible employment forms in community factories, to accurately reflect farmers’ livelihood strategies during this unique period. Additionally, based on sample characteristics, where the proportion of low-income rural households engaged in commercial and industrial self-employment is extremely low, this study does not consider self-employed industrial and commercial activities.

Within the above conceptual framework, this study operationalizes non-farm labor supply from two dimensions: the extensive margin and the intensive margin. On the extensive margin, the non-farm labor participation rate is used to measure households’ access to non-farm employment, defined as the proportion of household members participating in non-farm labor during the year relative to the total household labor force population. To test the localization bias hypothesis in the theoretical framework, the overall participation rate is further decomposed into the local non-farm labor participation rate, defined as the proportion of members participating in non-farm labor within the county, and the non-local non-farm labor participation rate, defined as the proportion of members participating in non-farm labor outside the county.

On the intensive margin, non-farm labor time is used to measure households’ intensity of non-farm employment input, defined as the total annual days household members engage in non-farm labor. Similarly, to reveal differences in employment location choice, this is decomposed into local non-farm labor time, defined as total days engaged in non-farm labor within the county, and non-local non-farm labor time, defined as total days engaged in non-farm labor outside the county. The original survey data used months as the time measurement unit. To improve research precision and enhance comparability with existing literature, this study adopts common methods to uniformly convert non-farm labor supply time into non-farm labor days, with 1 month equaling 30 days.

#### 4.2.2 Core explanatory variables

The core explanatory variable in this study is the Medical Financial Assistance policy, specifically referring to the cash compensation for medical expenses provided by the government to low-income rural households. This compensation occurs after households have incurred medical expenses and received reimbursements from basic medical insurance and catastrophic illness insurance according to regulations. This narrow definition excludes charitable assistance from non-governmental sources and also excludes the ex-ante premium subsidy component. It focuses on explicit policy interventions that directly alter households’ ex-post budget constraints and whose occurrence depends on exogenous health shocks, thereby providing a clearer causal pathway for identifying their economic behavioral effects. Based on this, this study quantifies the MFA policy from two dimensions.

##### 4.2.2.1 MFA receipt

This variable is a binary dummy variable indicating whether a low-income rural household actually received the previously mentioned ex-post expense compensation in the current year. If any household member received policy-compliant medical reimbursements and obtained assistance funds during the year, the variable is assigned a value of 1; otherwise, it is 0. This variable reflects the presence or absence of policy intervention and coverage breadth, and it is used to identify the basic effect of obtaining MFA eligibility.

##### 4.2.2.2 MFA intensity

This variable is a continuous measure of the total amount of MFA received by a low-income rural household in the current year, transformed by taking the natural logarithm to mitigate right-skewed distribution. This variable reflects the intensity of policy support and is used to test whether, within households that have already received assistance, increases in assistance amounts produce significant marginal incentive effects.

#### 4.2.3 Control variables

To control for potential confounding factors affecting non-farm labor supply, this study draws on labor supply theory (2, 3), health demand theory (1), and farm household decision models (76), and references existing research findings to introduce the following three categories of control variables.

##### 4.2.3.1 Household head characteristics

As the primary decision-maker, the household head’s personal characteristics influence household labor allocation. This study controls for the household head’s age and its square term to capture nonlinear life cycle effects. It also accounts for household head gender to reflect differences in social roles and resource endowments (77). Additionally, it controls for the household head’s years of education as a core measure of human capital and whether the household head is a Communist Party member to capture potential influences from social networks and information advantages. Although characteristics such as household head gender, education level, and political affiliation are theoretically stable, the large-scale tracking survey data used in this study confirm that these variables experienced small changes during the observation period due to household head replacement and information registration updates, with change proportions ranging from 0.73 percent to 1.05 percent. Incorporating these variables as time-varying controls in the model is necessary based on data facts and rigorous econometric principles.

##### 4.2.3.2 Household characteristics

This study controls for three key household structure variables. First, household size, measured by the number of co-residing members, directly affects the household’s labor pool and division of labor (78). Second, the health-adjusted labor ratio. To more precisely control for the constraint of health shocks on non-farm labor supply capacity, this study constructs this core variable, measured as follows:


$$\text{HALR}={\frac{0*N_{0}+0.5*N_{0.5}+1.0*N_{1}+1.5*{Ν}_{1.5}}{N}}^{*}100\%$$ (5)

In Equation 5, *HALR* represents the household health-adjusted labor ratio, *N* is the total number of benchmark labor force population aged 16 to 60 in the household, and *N_1.5_, N_1.0_, N_0.5_*, and *N_0_* are the numbers of skilled laborers, ordinary laborers, semi-laborers, and individuals with no labor capacity, respectively. The specific classification criteria follow the Guidelines for Dynamic Monitoring and Assistance to Prevent Return to Poverty issued by the National Rural Revitalization Administration, as shown in Table 2. By assigning differentiated weights of 0, 0.5, 1, and 1.5 to laborers with different health statuses, this indicator incorporates labor capacity impairments caused by illness, disability, and aging into the quantitative framework. It can measure the household’s actual available effective labor endowment more precisely than traditional labor force population ratios and serves as a key variable for controlling the potential confounding effects of health human capital on non-farm labor supply (79). Third, the household dependency ratio, defined as the proportion of members aged below 16 and above 60, measures household caregiving burdens and is expected to inhibit labor mobility (44).

**Table 2:** Calculation of health-adjusted labor force in low-income farm households.

Labor type	Criteria	Weight
No labor capacity or lost labor capacity	1. Minors aged below 16	0
	2. Disabled older adults aged above 60	
	3. Persons aged 16–60 who have lost labor capacity due to illness, and persons with first	
	or second degree physical, mental, or intellectual disabilities	
Weak labor or semi-labor	1. Ill persons aged 16–60 with labor capacity	0.5
	2. Healthy older adults aged above 60 capable of simple labor	
	3. Persons with partial labor capacity due to disability, assessed through villagers’ democratic appraisal	
Ordinary labor	Persons aged 16–60 with labor capacity but without professional qualification certificates	1
Skilled labor	Persons aged 16–60 with labor capacity and holding professional qualification certificates certified by human resources and social security departments	1.5

Diseases refer to the 30 major diseases specified in the Diseases for Special Treatment of Major Illnesses among the Poor Population issued by the National Health Commission and the four types of chronic diseases specified in the National Basic Public Health Service Standards (Third Edition), including hypertension, type 2 diabetes, tuberculosis, and severe mental disorders. The determination of labor capacity for persons with disabilities is assessed based on their actual situation in daily life and production, evaluated during villagers’ meetings, and verified by village committees. Except for persons with first or second degree physical disabilities, mental disabilities, and intellectual disabilities, adults with other types of disabilities are considered to have labor capacity. Household labor force information is obtained through questionnaire surveys and verified with village committee records. Medical diagnoses are based on certificates from county-level hospitals.

##### 4.2.3.3 Economic characteristics

This study controls for three variables characterizing household resource endowments and livelihood patterns. First, cultivated land area reflects the potential lock-in effect of land resources on agricultural labor. Second, the agricultural income share, defined as the ratio of agricultural income to total household income, measures the household’s dependence on agriculture and the opportunity cost of shifting labor to non-farm activities. Third, household per capita net income characterizes the household’s overall economic foundation and risk-bearing capacity, representing a key factor influencing non-farm labor supply decisions. Specific definitions and descriptive statistics for each variable are presented in Table 3.

**Table 3:** Variable definitions and descriptive statistics.

Variable type	Variable name	Definition or measurement method	Mean	Std. Dev.
Dependent variables				
Extensive margin	Overall NFPR	Proportion of household members participating in non-farm labor relative to the total household labor force	0.914	0.504
	Local NFPR	Proportion of members participating in non-farm labor within the county	0.807	0.557
	Non-local NFPR	Proportion of members participating in non-farm labor outside the county	0.103	0.245
Intensive margin	Overall NFLT	Total annual days household members engaged in non-farm labor	239.854	153.423
	Local NFLT	Total annual days engaged in non-farm labor within the county	204.761	141.623
	Non-local NFLT	Total annual days engaged in non-farm labor outside the county	33.962	80.560
Core explanatory variables	MFA receipt	Whether household received MFA during the year, yes = 1, no = 0	0.497	0.500
	MFA intensity	Log of annual MFA amount plus one	3.087	3.351
Household head characteristics	Age	Household head’s actual age in years	59.085	11.730
	Age squared	Household head’s age squared divided by 100	36.287	13.878
	Gender	Household head’s gender, male = 1, female = 0	0.856	0.351
	Education	Household head’s actual years of education	6.793	2.886
	Party membership	Whether the household head is a Party member, yes = 1, no = 0	0.055	0.229
Household characteristics	Household size	Number of co-residing members in the current year	2.673	1.378
	HALR	Health-adjusted labor ratio calculated using Equation 5	0.688	0.287
	Household dependency Ratio	(Population below 16 + population above 60)/total population	0.348	0.352
Economic characteristics	Cultivated land area	Household’s actual cultivated land area in mu	3.790	2.549
	Agricultural income share	Household agricultural income/total income	0.086	0.138
	Household per capita net income	Log of annual household per capita net income	9.205	0.271

NFPR denotes non-farm labor participation rate. NFLT denotes non-farm labor time measured in days. HALR denotes health-adjusted labor ratio. MFA denotes Medical Financial Assistance.

### 4.3 Model specification

#### 4.3.1 Baseline models

To accurately identify the causal effect of the MFA policy on the non-farm labor supply of low-income rural households, this study constructs two-way fixed effects models based on the two-wave panel data from 2021 to 2022. These models, by simultaneously controlling for household fixed effects and year fixed effects, effectively mitigate endogeneity biases arising from unobserved individual heterogeneity and common time trends. To comprehensively examine policy effects, this study constructs models from two dimensions: policy coverage breadth and policy support intensity.


$$\;\;Y_{\mathrm{it}}=\alpha_{0}+\beta D_{\mathrm{it}}+\gamma X_{\mathrm{it}}+\mu_{i}+\lambda_{t}+\varepsilon_{\mathrm{it}}\;\;$$ (6)

$$\;\;Y_{\mathrm{it}}=\alpha_{1}+\mathrm{\delta ln}A_{\mathrm{it}}+\phi X_{\mathrm{it}}+\nu_{i}+\varphi_{t}+\xi_{\mathrm{it}}$$ (7)

In Equations 6, 7, subscripts *i* and *t* denote household and year, respectively, with *t* representing 2021 or 2022. The dependent variable *Y_it_* encompasses both the extensive and intensive margins of non-farm labor supply, specifically including the overall, local, and non-local non-farm labor participation rates, as well as the overall, local, and non-local non-farm labor time measured in days.

Equation 6 represents the policy breadth effect model. The core explanatory variable *D_it_* is a binary dummy variable that takes the value of 1 if household *i* actually received MFA funds in year *t,* and 0 otherwise. Its coefficient β reflects the average treatment effect of obtaining MFA eligibility on non-farm labor supply after controlling for other factors, representing the eligibility effect.

Equation 7 represents the policy intensity effect model. The core explanatory variable *lnA_it_* is a continuous variable measured as the natural logarithm of the total annual MFA amount received by the household plus one. The coefficient δ reflects the marginal impact of a 1 % increase in assistance amounts on non-farm labor supply, representing the intensity effect.

$X_{\mathrm{it}}$ represents the vector of control variables. $\mu_{i}$ and $\nu_{i}$ represent household fixed effects, which absorb all time-invariant household heterogeneity, such as initial health endowments, geographic location, and long-term risk preferences. $\lambda_{t}$ and $\varphi_{t}$ represent year fixed effects, which control for common time shocks affecting all farm households between 2021 and 2022, particularly the recurring COVID-19 pandemic, macroeconomic fluctuations, and regional containment policies. $\varepsilon_{\mathrm{it}}$ and $\xi_{\mathrm{it}}$ are random disturbance terms. All estimations employ robust standard errors clustered at the village level to address potential correlations within villages.

By separately estimating models (6) and (7), this study can clearly distinguish and test the following aspects. First, whether obtaining MFA eligibility constitutes a critical threshold affecting non-farm labor supply, representing the eligibility effect. Second, within households that have already received assistance, whether increases in assistance amounts generate further marginal incentives, representing the intensity effect. This dual specification provides a comprehensive econometric foundation for testing the aforementioned research hypotheses, with results from model (6) serving as core evidence and results from model (7) used to reveal the dose–response characteristics of policy effects.

#### 4.3.2 Model specification tests

To statistically verify the appropriateness of model specifications, this study conducted rigorous Hausman tests for all baseline model specifications. The null hypothesis of the Hausman test is that the random effects model is appropriate, meaning that unobserved individual effects are uncorrelated with all explanatory variables. Rejection of the null hypothesis indicates that estimates from the random effects model may be biased and that the fixed effects model should be adopted.

The test results are presented in Table 4. Regardless of whether the dependent variable is the non-farm labor participation rate on the extensive margin or non-farm labor time on the intensive margin, and regardless of whether the core explanatory variable is MFA receipt or MFA intensity, all test statistics yield *p*-values of 0.0000, strongly rejecting the null hypothesis at the 1 % significance level. This indicates that the random effects model specification is inappropriate for all scenarios in this study. Therefore, adopting the two-way fixed effects model as the baseline regression model is necessary and reliable from a statistical perspective.

**Table 4:** Model specification tests (Hausman Tests).

Dependent variable	Core explanatory variable	*χ* ^2^	*p*-value
Non-farm labor participation rate (extensive margin)			
Overall NFPR	Receipt (*D_it_*)	255.65	0.0000
	Intensity (*lnA_it_*)	255.94	0.0000
Local NFPR	Receipt (*D_it_*)	239.13	0.0000
	Intensity (*lnA_it_*)	240.43	0.0000
Non-local NFPR	Receipt (*D_it_*)	141.61	0.0000
	Intensity (*lnA_it_*)	142.62	0.0000
Non-farm labor time (intensive margin)			
Overall NFLT	Receipt (*D_it_*)	124.31	0.0000
	Intensity (l*nA_it_*)	126.52	0.0000
Local NFLT	Receipt (*D_it_*)	45.40	0.0000
	Intensity (*lnA_it_*)	50.27	0.0000
Non-local NFLT	Receipt (*D_it_*)	195.74	0.0000
	Intensity (*lnA_it_*)	195.17	0.0000

NFPR denotes non-farm labor participation rate. NFLT denotes non-farm labor time measured in days. MFA denotes Medical Financial Assistance. All estimations employ robust standard errors clustered at the village level.

## 5 Results

### 5.1 Sample characteristics and potential selection bias analysis

To reveal the sample structure and the targeting of the Medical Financial Assistance policy, and to highlight the importance of subsequent causal identification, this study conducts grouped descriptions and comparisons based on whether low-income rural households received MFA. Table 5 reports the descriptive statistics and group mean *t*-test results by MFA receipt status.

**Table 5:** Comparison of sample characteristics by MFA status.

Variable	Receiving MFA		Not receiving MFA		Group difference
	Mean	SD	Mean	SD	
Non-farm labor supply					
Overall NFPR	0.896	0.505	0.932	0.503	−0.036***
Local NFPR	0.796	0.551	0.819	0.563	−0.024***
Non-local NFPR	0.098	0.232	0.108	0.257	−0.010***
Overall NFLT (days)	248.261	160.060	231.539	146.095	16.721***
Local NFLT (days)	212.470	146.529	197.136	136.176	15.334***
Non-local NFLT (days)	34.531	81.360	33.400	79.761	1.131
Household head and household characteristics					
Household head age (years)	60.411	11.475	57.775	11.833	2.636***
Household head age squared/100	37.811	13.907	34.779	13.683	3.032***
Household head male (=1)	0.868	0.338	0.845	0.362	0.023***
Household head education (years)	6.829	2.873	6.758	2.899	0.071**
Household head party member	0.068	0.252	0.043	0.202	0.025***
Household size (persons)	2.896	1.397	2.454	1.321	0.442***
HALR	0.659	0.280	0.717	0.292	−0.058***
Household dependency ratio	0.375	0.349	0.322	0.353	0.053***
Economic characteristics					
Cultivated land area (mu)	4.012	2.603	3.570	2.475	0.442***
Agricultural income share	0.082	0.133	0.090	0.142	−0.008***
*Per Capita* net income (log)	9.173	0.243	9.236	0.293	−0.063***
Number of households	13,024		13,169		

MFA denotes Medical Financial Assistance. NFPR denotes non-farm labor participation rate. NFLT denotes non-farm labor time. HALR denotes health-adjusted labor ratio. SD denotes standard deviation. *, **, and *** denote significance at the 10, 5, and 1% levels, respectively. Group difference is calculated as the mean of the recipient group minus the mean of the non-recipient group.

Group comparisons show that on the extensive margin of non-farm labor supply, households receiving MFA are at a significant disadvantage. Their overall non-farm labor participation rate is 0.896, which is 3.6 percentage points lower than that of non-recipient households (0.932). Local and non-local non-farm labor participation rates are also significantly lower by 2.4 and 1.0 percentage points, respectively. However, on the intensive margin, recipient households have an overall non-farm labor time of 248.26 days, which is 16.72 days more than non-recipient households. This difference is primarily driven by increases in local non-farm labor time. The coexistence of disadvantages on the extensive margin and advantages on the intensive margin reveals the complex decision-making logic of vulnerable households under health constraints and livelihood pressures. Health shocks may raise the threshold for entering the labor market, inhibiting participation, but heavy economic burdens force households to mobilize available labor resources to the maximum extent, extending non-farm labor time to maintain livelihoods and thereby intensifying labor input.

In terms of other characteristics, the systematic vulnerability of recipient households is equally pronounced. Their health human capital is significantly impaired, with the health-adjusted labor ratio 5.8 percentage points lower than that of non-recipient households, at 0.659 compared to 0.717. Their caregiving burden is heavier, with the dependency ratio 5.3 percentage points higher, at 0.375 compared to 0.322. Economically, although their cultivated land area is 0.44 mu larger, their agricultural income-generating capacity is weaker, with the agricultural income share 0.8 percentage points lower, and their per capita net income significantly lower by 0.063 units. These characteristics collectively constitute the initial conditions of households before policy intervention and represent sources of selection bias that make them more likely to receive assistance.

The above results clearly confirm the targeting of the MFA policy in practice, namely that it precisely covers groups that are systematically disadvantaged in terms of health, household burden, and economic status. However, all associations revealed in Table 5, whether concerning disadvantages in non-farm labor participation rates or the reverse differences in non-farm labor time, reflect the initial conditions and selection bias of the sample before policy intervention, rather than the causal effects of the policy intervention. This simple cross-sectional comparison has fundamental limitations. It cannot distinguish between households’ inherent vulnerability and the impact of policy intervention. Therefore, any associations observed in the table are essentially residual effects of negative selection factors such as health shocks or manifestations of vulnerable households’ initial decisions and cannot be directly interpreted as causal effects of the policy.

Given this, to isolate the endogeneity bias caused by initial disadvantages and time-invariant heterogeneity and to accurately identify the net effect of MFA, this study employs two-way fixed effects models in the following analysis. These models, by controlling for household fixed effects to absorb all time-invariant household heterogeneity and year fixed effects to filter out common macro trends, can effectively estimate the causal effects of the policy.

### 5.2 Baseline regression results

#### 5.2.1 Impact of Medical Financial Assistance on non-farm labor participation rate

Table 6 reports the baseline estimation results of the impact of MFA on the non-farm labor participation rate of low-income rural households. Columns (1) to (3) use MFA receipt as the core explanatory variable to test hypothesis H1a. Columns (4) to (6) use the logarithm of the MFA amount as the core explanatory variable to test hypothesis H1b.

**Table 6:** Benchmark regression: impact of MFA on NFPR.

Variable	Overall NFPR	Local NFPR	Non-local NFPR	Overall NFPR	Local NFPR	Non-local NFPR
	(1)	(2)	(3)	(4)	(5)	(6)
MFA receipt	0.020*** (0.007)	0.017** (0.007)	0.002 (0.002)			
MFA amount (log)				0.002** (0.001)	0.002 (0.001)	0.000 (0.000)
Household head age	−0.033* (0.017)	−0.024 (0.017)	−0.010** (0.004)	−0.034* (0.017)	−0.024 (0.017)	−0.011** (0.004)
Household head age^2^	0.000* (0.000)	0.000 (0.000)	0.000** (0.000)	0.000* (0.000)	0.000 (0.000)	0.000** (0.000)
Household head gender	0.040 (0.067)	0.089 (0.067)	−0.039** (0.016)	0.040 (0.067)	0.089 (0.066)	−0.039** (0.016)
Household head education	0.003 (0.014)	0.007 (0.014)	−0.005 (0.004)	0.003 (0.014)	0.007 (0.014)	−0.005 (0.004)
Household head party member	−0.030 (0.033)	−0.016 (0.037)	−0.016 (0.014)	−0.030 (0.033)	−0.016 (0.037)	−0.016 (0.014)
Household size	0.002 (0.017)	−0.019 (0.019)	0.025** (0.011)	0.002 (0.017)	−0.018 (0.019)	0.024** (0.011)
HALR	0.590*** (0.092)	0.564*** (0.093)	0.020* (0.011)	0.590*** (0.092)	0.564*** (0.093)	0.020* (0.011)
Household dependency ratio	0.196* (0.100)	0.267** (0.112)	−0.094** (0.042)	0.196* (0.100)	0.267** (0.113)	−0.095** (0.042)
Cultivated land area	0.029*** (0.010)	0.061*** (0.012)	−0.033*** (0.006)	0.029*** (0.010)	0.061*** (0.012)	−0.033*** (0.006)
Agricultural income share	−0.049 (0.078)	−0.016 (0.077)	−0.030** (0.015)	−0.049 (0.078)	−0.015 (0.077)	−0.030** (0.015)
Per capita net income	0.173*** (0.033)	0.149*** (0.037)	0.019 (0.013)	0.172*** (0.033)	0.148*** (0.037)	0.019 (0.013)
Constant	−0.356 (0.657)	−0.673 (0.666)	0.386** (0.168)	−0.341 (0.658)	−0.663 (0.667)	0.390** (0.169)
Household FE	YES	YES	YES	YES	YES	YES
Year FE	YES	YES	YES	YES	YES	YES
*R* ^2^	0.082	0.079	0.012	0.082	0.078	0.012
*N*	26,193	26,193	26,193	26,193	26,193	26,193

MFA denotes Medical Financial Assistance. NFPR denotes non-farm labor participation rate. HALR denotes health-adjusted labor ratio. FE denotes fixed effects. Clustered robust standard errors are in parentheses. *, **, and *** denote significance at the 10, 5, and 1% levels, respectively. Household per capita net income is log-transformed.

From the estimation results in columns (1) to (3), obtaining MFA eligibility has a significant positive impact on the non-farm labor participation rate of low-income rural households. After controlling for household fixed effects and year fixed effects, obtaining assistance increases the household’s overall non-farm labor participation rate by 2.0 percentage points (*β* = 0.020, *p* < 0.01). Further decomposition reveals that this effect is primarily concentrated in the local market: The local non-farm labor participation rate increases by 1.7 percentage points (*β* = 0.017, *p* < 0.05), while the non-local non-farm labor participation rate increases by only 0.2 percentage points (*β* = 0.002, *p* > 0.1), which does not pass the significance test. This result supports hypothesis H1a, indicating that obtaining MFA eligibility has a significant stimulating effect on the non-farm labor participation decisions of low-income rural households, and this effect is localized.

Columns (4) to (6) examine the marginal impact of assistance amounts. The results show that increases in assistance amounts have a very limited incentive effect on the non-farm labor participation rate of low-income rural households. A one-unit increase in the logarithm of the assistance amount increases the overall non-farm labor participation rate by only 0.002 percentage points (*β* = 0.002, *p* < 0.05), significant at the 5% level. For the local non-farm labor participation rate, the estimated coefficient for the logarithm of the assistance amount is 0.002 (*p* > 0.1); for the non-local non-farm labor participation rate, the estimated coefficient is 0.000 (*p* > 0.1), neither of which passes the significance test. This result supports hypothesis H1b, indicating that increases in MFA amounts have no significant effect on the non-farm labor participation rate, and the incentive effect of the policy is primarily manifested through obtaining eligibility, representing a threshold effect.

#### 5.2.2 Impact of Medical Financial Assistance on non-farm labor time

Table 7 reports the baseline estimation results of the impact of MFA on the non-farm labor time of low-income rural households. Columns (1) to (3) use MFA receipt as the core explanatory variable to test hypothesis H2a. Columns (4) to (6) use the logarithm of the MFA amount as the core explanatory variable to test hypothesis H2b.

**Table 7:** Benchmark regression: impact of MFA on NFLT.

Variable	Overall NFLT	Local NFLT	Non-local NFLT	Overall NFLT	Local NFLT	Non-local NFLT
	(1)	(2)	(3)	(4)	(5)	(6)
MFA receipt	4.871*** (1.781)	4.560*** (1.723)	0.340 (0.701)			
MFA amount (log)				0.312 (0.254)	0.318 (0.251)	0.016 (0.113)
Household head age	5.840 (3.813)	4.331 (3.468)	1.470 (1.448)	5.829 (3.813)	4.313 (3.464)	1.471 (1.449)
Household head age^2^	−0.051 (0.031)	−0.037 (0.028)	−0.013 (0.011)	−0.051 (0.031)	−0.037 (0.028)	−0.013 (0.011)
Household head gender	34.036*** (10.990)	41.927*** (10.942)	−8.842** (3.843)	34.164*** (10.982)	42.022*** (10.936)	−8.828** (3.829)
Household head education	−3.308 (2.604)	−2.304 (2.595)	−0.831 (0.670)	−3.334 (2.605)	−2.325 (2.596)	−0.833 (0.670)
Household head party member	−9.967 (11.976)	−6.805 (10.978)	−2.973 (3.795)	−9.859 (11.880)	−6.714 (10.886)	−2.963 (3.795)
Household size	50.242*** (4.736)	34.972*** (5.152)	14.639*** (3.039)	50.395*** (4.752)	35.098*** (5.168)	14.653*** (3.045)
HALR	145.479*** (14.308)	128.136*** (14.325)	16.904*** (2.876)	145.585*** (14.319)	128.236*** (14.334)	16.911*** (2.880)
Household dependency ratio	−32.063 (23.184)	−5.479 (23.475)	−24.795*** (9.291)	−31.768 (23.145)	−5.245 (23.463)	−24.766*** (9.274)
Cultivated land area	2.743 (10.903)	4.568 (11.087)	−1.558 (1.247)	2.701 (10.865)	4.532 (11.056)	−1.562 (1.248)
Agricultural income share	−17.496 (17.053)	−8.842 (16.921)	−9.405** (4.226)	−17.347 (17.075)	−8.709 (16.940)	−9.393** (4.226)
Per capita net income	41.394*** (13.010)	36.720*** (12.320)	4.118 (3.152)	41.309*** (13.026)	36.640*** (12.336)	4.112 (3.150)
Constant	−562.5*** (184.654)	−492.5*** (173.533)	−66.674 (52.495)	−560.7*** (184.797)	−490.6*** (173.554)	−66.594 (52.497)
Household FE	YES	YES	YES	YES	YES	YES
Year FE	YES	YES	YES	YES	YES	YES
*R* ^2^	0.223	0.191	0.014	0.223	0.191	0.014
*N*	26,193	26,193	26,193	26,193	26,193	26,193

MFA denotes Medical Financial Assistance. NFLT denotes non-farm labor time measured in days. HALR denotes health-adjusted labor ratio. FE denotes fixed effects. Clustered robust standard errors are in parentheses. *, **, and *** denote significance at the 10, 5, and 1% levels, respectively.

Columns (1) to (3) show that obtaining MFA eligibility also significantly increases the non-farm labor time input of low-income rural households. Obtaining assistance increases the overall non-farm labor time of low-income rural households by 4.87 days (*β* = 4.871, *p* < 0.01). Among this, local non-farm labor time increases by 4.56 days (*β* = 4.560, *p* < 0.01), accounting for 93.6% of the total increase. Non-local non-farm labor time increases by 0.34 days (*β* = 0.340, *p* > 0.1), which does not pass the significance test. This pattern is highly consistent with the findings in the participation rate dimension, supporting hypothesis H2a.

Columns (4) to (6) show that increases in assistance amounts do not lead to significant extensions in non-farm labor time. A one-unit increase in the logarithm of the assistance amount yields an estimated coefficient of 0.312 days for overall non-farm labor time (*β* = 0.312, *p* > 0.1), 0.318 days for local non-farm labor time (*β* = 0.318, *p* > 0.1), and 0.016 days for non-local non-farm labor time (*β* = 0.016, *p* > 0.1). None of these coefficients pass the significance test at the 10% level. This result supports hypothesis H2b, further confirming the threshold characteristic of the policy effect.

In summary, the baseline regression analysis establishes the causal empowering effect of the MFA policy on the non-farm labor supply of low-income rural households. However, this effect is subject to two structural constraints. First, its impact has a distinct geographical boundary, manifested as a significant promotion of local non-farm employment but limited effects on non-local mobility. Second, its effect has a clear policy threshold, manifested as a significant effect triggered by obtaining eligibility but weak marginal incentives from increasing amounts. These findings provide key empirical evidence for accurately evaluating and optimizing the developmental function of the MFA policy.

### 5.3 Robustness checks

#### 5.3.1 Winsorization test

To exclude the potential interference of extreme observations on the estimation results, this study applies winsorization at the 1st and 99th percentiles to all continuous variables in the full sample and re-estimates the baseline models.

Table 8 reports the estimation results of the impact of MFA on the non-farm labor participation rate of low-income rural households after winsorization. Columns (1) to (3) show that the promoting effect of obtaining MFA on the non-farm labor participation rate remains robust. Obtaining assistance significantly increases the household’s overall non-farm labor participation rate by 1.9 percentage points (*β* = 0.019, *p* < 0.01), the local non-farm labor participation rate by 1.7 percentage points (*β* = 0.017, *p* < 0.05), while the impact on the non-local non-farm labor participation rate remains insignificant (*β* = 0.002, *p* > 0.1). These results are highly consistent with the baseline regression in terms of coefficient magnitude and significance levels. Columns (4) to (6) further confirm the threshold effect. After excluding extreme values, a one-unit increase in the logarithm of the assistance amount has a weak but significant positive impact on the overall non-farm labor participation rate (*β* = 0.002, *p* < 0.05), while the impacts on local (*β* = 0.002, *p* > 0.1) and non-local (*β* = 0.000, *p* > 0.1) non-farm labor participation rates do not pass the significance test.

**Table 8:** Robustness check (winsorization): impact of MFA on NFPR.

Variable	Overall NFPR	Local NFPR	Non-local NFPR	Overall NFPR	Local NFPR	Non-local NFPR
	(1)	(2)	(3)	(4)	(5)	(6)
MFA receipt	0.019*** (0.007)	0.017** (0.008)	0.002 (0.002)			
MFA amount (log)				0.002** (0.001)	0.002 (0.001)	0.000 (0.000)
Controls	YES	YES	YES	YES	YES	YES
Constant	−0.278 (0.658)	−0.582 (0.669)	0.376** (0.172)	−0.262 (0.659)	−0.570 (0.670)	0.380** (0.172)
Household FE	YES	YES	YES	YES	YES	YES
Year FE	YES	YES	YES	YES	YES	YES
*R* ^2^	0.083	0.080	0.012	0.083	0.079	0.012
*N*	25,701	25,701	25,701	25,701	25,701	25,701

MFA denotes Medical Financial Assistance. NFPR denotes non-farm labor participation rate. FE denotes fixed effects. All continuous variables are winsorized at the 1st and 99th percentiles. Clustered robust standard errors are in parentheses. *, **, and *** denote significance at the 10, 5, and 1% levels, respectively.

Table 9 presents the winsorization test results for the non-farm labor time dimension. Columns (1) and (2) show that the positive impact of obtaining MFA on overall and local non-farm labor time remains robust, with coefficients of 4.727 (*p* < 0.01) and 4.373 (*p* < 0.01), respectively. Column (3) shows that the impact on non-local non-farm labor time remains insignificant (*β* = 0.361, *p* > 0.1). Columns (4) to (6) further reinforce the limited role of policy intensity. The coefficients for the impact of a one-unit increase in the logarithm of the assistance amount on overall, local, and non-local non-farm labor time are all insignificant (*β* = 0.322, *p* > 0.1; *β* = 0.323, *p* > 0.1; *β* = 0.017, *p* > 0.1, respectively). These results indicate that the core conclusions remain robust after excluding extreme values.

**Table 9:** Robustness check (winsorization): impact of MFA on NFLT.

Variable	Overall NFLT	Local NFLT	Non-local NFLT	Overall NFLT	Local NFLT	Non-local NFLT
	(1)	(2)	(3)	(4)	(5)	(6)
MFA receipt	4.727*** (1.752)	4.373*** (1.677)	0.361 (0.725)			
MFA amount (log)				0.322 (0.252)	0.323 (0.246)	0.017 (0.117)
Controls	YES	YES	YES	YES	YES	YES
Constant	−542.5*** (185.291)	−469.8*** (173.785)	−70.813 (52.899)	−540.6*** (185.445)	−467.7*** (173.814)	−70.720 (52.892)
Household FE	YES	YES	YES	YES	YES	YES
Year FE	YES	YES	YES	YES	YES	YES
*R* ^2^	0.222	0.191	0.014	0.222	0.190	0.014
*N*	25,701	25,701	25,701	25,701	25,701	25,701

MFA denotes Medical Financial Assistance. NFLT denotes non-farm labor time measured in days. FE denotes fixed effects. All continuous variables are winsorized at the 1st and 99th percentiles. Clustered robust standard errors are in parentheses. *, **, and *** denote significance at the 10, 5, and 1% levels, respectively.

#### 5.3.2 Controlling for potential omitted variables

Although the baseline models already control for household fixed effects and year fixed effects, industrial development at the village level could simultaneously affect MFA accessibility and the creation of non-farm employment opportunities, potentially confounding the estimation of the policy’s net effect. To address this potential omitted variable bias, this article introduces three variables reflecting village-level industrial driving capacity into the baseline models. These variables include whether the household is associated with a leading enterprise, whether it has joined a farmer professional cooperative, and whether it is led by an entrepreneurship-driven leader.

Table 10 reports the estimation results of the impact of MFA on the non-farm labor participation rate after controlling for village-level variables. Columns (1) to (3) show that after controlling for village-level variables, obtaining MFA still significantly promotes the overall non-farm labor participation rate (*β* = 0.020, *p* < 0.01) and the local non-farm labor participation rate (*β* = 0.018, *p* < 0.05), while the impact on the non-local non-farm labor participation rate remains insignificant (*β* = 0.002, *p* > 0.1). Columns (4) to (6) show that a one-unit increase in the logarithm of the assistance amount only weakly improves the overall non-farm labor participation rate (*β* = 0.002, *p* < 0.05), while the impacts on local and non-local non-farm labor participation rates are both insignificant (*β* = 0.002, *p* > 0.1; *β* = 0.000, *p* > 0.1, respectively).

**Table 10:** Robustness check (additional controls): impact of MFA on NFPR.

Variable	Overall NFPR	Local NFPR	Non-local NFPR	Overall NFPR	Local NFPR	Non-local NFPR
	(1)	(2)	(3)	(4)	(5)	(6)
MFA receipt	0.020*** (0.007)	0.018** (0.007)	0.002 (0.002)			
MFA amount (log)				0.002** (0.001)	0.002 (0.001)	0.000 (0.000)
Controls	YES	YES	YES	YES	YES	YES
Constant	−0.302 (0.657)	−0.615 (0.666)	0.383** (0.168)	−0.287 (0.658)	−0.604 (0.667)	0.387** (0.168)
Household FE	YES	YES	YES	YES	YES	YES
Year FE	YES	YES	YES	YES	YES	YES
*R* ^2^	0.082	0.079	0.012	0.082	0.078	0.012
*N*	26,193	26,193	26,193	26,193	26,193	26,193

MFA denotes Medical Financial Assistance. NFPR denotes non-farm labor participation rate. FE denotes fixed effects. Additional controls include three village-level industrial driving capacity variables. Clustered robust standard errors are in parentheses. *, **, and *** denote significance at the 10, 5, and 1% levels, respectively.

Table 11 reports the estimation results of the impact of MFA on non-farm labor time after controlling for village-level variables. Columns (1) to (3) show that the positive impact of obtaining MFA on overall and local non-farm labor time remains strong, with coefficients of 4.842 (*p* < 0.01) and 4.547 (*p* < 0.01), respectively, while the impact on non-local non-farm labor time remains insignificant (*β* = 0.325, *p* > 0.1). Columns (4) to (6) indicate that the estimated coefficients for MFA intensity are all insignificant, with a one-unit increase in the logarithm of the assistance amount yielding coefficients of 0.305 (*p* > 0.1), 0.316 (*p* > 0.1), and 0.012 (*p* > 0.1) for overall, local, and non-local non-farm labor time, respectively. These results indicate that the localization preference and threshold effect revealed in the baseline regression are not attributable to the confounding factor of uneven village-level industrial development, and the results are highly robust.

**Table 11:** Robustness check (additional controls): impact of MFA on NFLT.

Variable	Overall NFLT	Local NFLT	Non-local NFLT	Overall NFLT	Local NFLT	Non-local NFLT
	(1)	(2)	(3)	(4)	(5)	(6)
MFA receipt	4.842*** (1.780)	4.547*** (1.723)	0.325 (0.702)			
MFA amount (log)				0.305 (0.254)	0.316 (0.251)	0.012 (0.114)
Controls	YES	YES	YES	YES	YES	YES
Constant	−567.9*** (185.833)	−498.7*** (174.878)	−65.991 (52.782)	−566.23*** (185.965)	−496.96*** (174.895)	−65.948 (52.796)
Household FE	YES	YES	YES	YES	YES	YES
Year FE	YES	YES	YES	YES	YES	YES
*R* ^2^	0.223	0.191	0.014	0.223	0.191	0.014
*N*	26,193	26,193	26,193	26,193	26,193	26,193

MFA denotes Medical Financial Assistance. NFLT denotes non-farm labor time measured in days. FE denotes fixed effects. Additional controls include three village-level industrial driving capacity variables. Clustered robust standard errors are in parentheses. *, **, and *** denote significance at the 10, 5, and 1% levels, respectively.

#### 5.3.3 Entropy balancing matching with difference-in-differences

To further address potential time-varying selection bias from observable household characteristics, this study employs entropy balancing matching combined with difference-in-differences estimation. Entropy balancing constructs weights for control households so that the covariate distributions of the treatment and control groups are exactly balanced in the pre-intervention period without discarding any observations. This method preserves the full sample and improves estimation efficiency.

The covariates used for balancing include household head characteristics such as age, age squared, gender, years of education, and party membership. Household structure variables include household size, health-adjusted labor share, dependency ratio, and illness share. Economic and health baseline variables include cultivated land area, baseline out-of-pocket medical expenses, employment assistance, and industrial assistance. All covariates are measured in the baseline year 2021. The treatment group consists of households that received MFA for the first time in 2022, while the control group consists of households that did not receive MFA in either 2021 or 2022.

The procedure involves two steps. First, entropy balancing weights are computed to achieve identical first moments of all covariates between the treatment and control groups. Second, the estimated weights are applied to the two-wave panel data, and a two-way fixed effects DID model is re-estimated on the full sample. The balancing diagnostics confirm that after weighting, all covariate means are well aligned between the two groups.

Table 12 reports the entropy balancing DID results. For non-farm labor participation, the effect of MFA receipt remains positive and significant. The overall participation rate increases by 3.5 percentage points, and the local participation rate increases by 2.9 percentage points. For non-farm labor time, the overall labor time increases by 6.35 days, and the local labor time increases by 5.78 days. The effects on non-local outcomes are not significant. These results confirm that the main conclusions are robust against selection bias based on observable characteristics.

**Table 12:** Robustness check: entropy balancing DID estimates for MFA receipt.

Variable	Overall NFPR	Local NFPR	Non-local NFPR	Overall NFLT	Local NFLT	Non-local NFLT
MFA receipt	0.035*** (0.012)	0.029** (0.012)	0.005 (0.003)	6.348** (3.224)	5.784* (3.093)	0.948 (1.052)
Control variables	YES	YES	YES	YES	YES	YES
Household FE	YES	YES	YES	YES	YES	YES
Year FE	YES	YES	YES	YES	YES	YES
Observations	25,356	25,356	25,356	25,356	25,356	25,356
*R* ^2^	0.077	0.075	0.011	0.220	0.189	0.014

MFA denotes Medical Financial Assistance. NFPR denotes non-farm labor participation rate. NFLT denotes non-farm labor time measured in days. FE denotes fixed effects. Entropy balancing weights are based on baseline covariates. Clustered robust standard errors at the village level are in parentheses. *, **, and *** denote significance at the 10, 5, and 1% levels, respectively.

### 5.4 Heterogeneity analysis

#### 5.4.1 Heterogeneity based on family life cycle

The family life cycle determines the internal structure of labor endowments and the allocation of caregiving responsibilities within the household. According to household production theory, older adult households typically face greater health risks and caregiving burdens, while prime-age households possess more abundant labor resources and greater flexibility in time allocation (2, 3). Therefore, the MFA policy may exhibit varied effects across households at different life cycle stages. This section aims to test hypotheses H3a and H4a proposed in the theoretical analysis.

To examine the heterogeneity of MFA policy effects by family life cycle, this study divides the sample into three categories based on the household’s demographic age structure: Youth households primarily focused on raising minors, middle-aged households primarily composed of prime-age laborers, and older adult households primarily burdened with older adult care. The classification method calculates the proportions of older adult members (aged 65 and above), prime-age members (aged 18 to 64), and minor members (aged below 18) in the household, categorizing it based on the member type with the highest proportion.

(1) *Heterogeneous impact on non-farm labor participation rate*. Table 13 reports the estimation results of the impact of MFA on the non-farm labor participation rate of low-income rural households across different family life cycles. Panel A uses MFA receipt as the core explanatory variable to test the heterogeneity of the policy breadth effect, corresponding to hypotheses H1a and H3a. Panel B uses the logarithm of the MFA amount as the core explanatory variable to test the heterogeneity of the policy intensity effect, corresponding to hypothesis H1c.

**Table 13:** Heterogeneity analysis by family life cycle: impact of MFA on NFPR.

Variable	Overall NFPR			Local NFPR			Non-local NFPR		
	Youth	Middle-aged	Older adults	Youth	Middle-aged	Older adults	Youth	Middle-aged	Older adults
	(1)	(2)	(3)	(4)	(5)	(6)	(7)	(8)	(9)
Panel A	MFA breadth								
MFA receipt	0.015* (0.008)	0.013* (0.008)	0.036* (0.019)	0.017* (0.010)	0.013 (0.008)	0.027 (0.019)	−0.001 (0.006)	0.001 (0.004)	0.005* (0.003)
Constant	2.364*** (0.637)	2.047** (0.862)	−3.239 (2.601)	1.186* (0.691)	1.888** (0.831)	−3.603 (2.655)	1.146** (0.523)	0.191 (0.263)	0.255 (0.426)
Wald test		2.92** (0.035)			2.52* (0.058)			1.09 (0.356)	
Panel B	MFA intensity								
MFA amount (log)	0.002* (0.001)	0.002 (0.001)	0.004 (0.003)	0.001 (0.001)	0.002 (0.001)	0.003 (0.003)	0.001 (0.001)	0.000 (0.001)	0.001 (0.000)
Constant	2.374*** (0.639)	2.055** (0.861)	−3.153 (2.600)	1.174* (0.694)	1.891** (0.829)	−3.542 (2.657)	1.169** (0.525)	0.196 (0.264)	0.269 (0.428)
Wald test		1.38 (0.250)			0.79 (0.503)			1.02 (0.384)	
Controls	YES	YES	YES	YES	YES	YES	YES	YES	YES
Household FE	YES	YES	YES	YES	YES	YES	YES	YES	YES
Year FE	YES	YES	YES	YES	YES	YES	YES	YES	YES
*R* ^2^	0.156	0.052	0.169	0.125	0.054	0.165	0.017	0.016	0.019
*N*	4,827	13,880	7,486	4,827	13,880	7,486	4,827	13,880	7,486

MFA denotes Medical Financial Assistance. NFPR denotes non-farm labor participation rate. FE denotes fixed effects. Youth, middle-aged, and older adult households are classified based on the highest proportion of members aged <18, 18–64, and ≥65, respectively. Wald tests examine inter-group differences in coefficients. Clustered robust standard errors are in parentheses. *, **, and *** denote significance at the 10, 5, and 1% levels, respectively.

From the estimation results in Panel A, obtaining MFA eligibility has a significant positive impact on the non-farm labor participation rate for all three types of households, but the magnitude of the effect varies. Regarding the overall non-farm labor participation rate, column (3) shows that the increase is largest for older adult households (*β =* 0.036, *p* < 0.1), column (1) shows that youth households have the next largest increase (*β* = 0.015, *p* < 0.1), and column (2) shows that middle-aged households have the smallest increase (*β* = 0.013, *p* < 0.1). Wald tests indicate that the inter-group difference in the overall non-farm labor participation rate is significant at the 5% level (*F* = 2.92, *p* = 0.035). Regarding employment location, the promoting effect on youth households is primarily manifested in the local market. Column (4) shows that the local non-farm labor participation rate increases by 1.7 percentage points (*β* = 0.017, *p* < 0.1). For older adult households, there is also a weak but significant increase in the non-local non-farm labor participation rate, with column (9) showing an estimated coefficient of 0.005 (*β* = 0.005, *p* < 0.1). These results validate hypothesis H3a, indicating that the promoting effect of MFA on the non-farm labor participation rate is most significant in older adult households with heavier caregiving burdens.

Panel B reports the estimation results for MFA intensity. Regarding the overall non-farm labor participation rate, column (1) shows that the coefficient for the logarithm of the assistance amount in youth households is significantly positive at the 10% level (*β* = 0.002, *p* < 0.1), while the coefficients for middle-aged households in column (2) and older adult households in column (3) do not reach significance. For local and non-local non-farm labor participation rates, the coefficients for the logarithm of the assistance amount are insignificant for all household types. Wald tests indicate that inter-group differences in the assistance amount coefficients are not significant for any of the dependent variables (*p* > 0.1). These results further confirm the universal judgment of hypothesis H1c regarding the threshold effect, namely that increases in assistance amounts do not generate significant marginal incentives for any household type.

(2) *Heterogeneous impact on non-farm labor time*. Table 14 reports the estimation results of the impact of MFA on non-farm labor time across different family life cycles. Panel A tests the heterogeneity of the policy breadth effect, corresponding to hypotheses H2a and H4a. Panel B tests the heterogeneity of the policy intensity effect, corresponding to hypothesis H2c.

**Table 14:** Heterogeneity analysis by family life cycle: impact of MFA on NFLT.

Variable	Overall NFLT			Local NFLT			Non-local NFLT		
	Youth	Middle-aged	Older adult Households	Youth	Middle-aged	Older adults	Youth	Middle-aged	Older adults
	(1)	(2)	(3)	(4)	(5)	(6)	(7)	(8)	(9)
Panel A	MFA breadth								
MFA receipt	6.491** (3.250)	5.563** (2.338)	2.404 (3.361)	5.696 (3.731)	4.983** (2.321)	2.208 (3.356)	0.162 (1.770)	0.814 (1.247)	0.245 (0.652)
Constant	417.288 (278.782)	−435.052* (258.770)	−731.881 (578.159)	256.279 (279.607)	−200.197 (257.554)	−784.543 (573.006)	72.062 (117.533)	−224.440*** (86.197)	57.459 (96.442)
Wald test		2.87** (0.037)			2.51* (0.059)			0.45 (0.715)	
Panel B	MFA intensity								
MFA amount (log)	0.972* (0.565)	0.405 (0.352)	−0.036 (0.460)	0.701 (0.641)	0.441 (0.367)	−0.072 (0.452)	0.168 (0.291)	0.037 (0.206)	0.040 (0.105)
Constant	424.649 (279.321)	−438.497* (258.562)	−733.329 (577.701)	259.117 (279.982)	−201.937 (257.194)	−786.743 (572.747)	75.674 (117.351)	−225.318*** (86.440)	58.275 (96.766)
Wald test		0.83 (0.476)			0.74 (0.531)			0.19 (0.900)	
Controls	YES	YES	YES	YES	YES	YES	YES	YES	YES
Household FE	YES	YES	YES	YES	YES	YES	YES	YES	YES
Year FE	YES	YES	YES	YES	YES	YES	YES	YES	YES
*R* ^2^	0.240	0.234	0.211	0.203	0.187	0.205	0.012	0.033	0.008
*N*	4,827	13,880	7,486	4,827	13,880	7,486	4,827	13,880	7,486

MFA denotes Medical Financial Assistance. NFLT denotes non-farm labor time measured in days. FE denotes fixed effects. Youth, middle-aged, and older adult households are classified based on the highest proportion of members aged <18, 18–64, and ≥65, respectively. Wald tests examine inter-group differences in coefficients. Clustered robust standard errors are in parentheses. *, **, and *** denote significance at the 10, 5, and 1% levels, respectively.

Panel A shows that the impact pattern of obtaining MFA eligibility on labor time contrasts sharply with that on the participation rate. Regarding overall non-farm labor time, column (1) indicates that obtaining MFA increases labor time for youth households by 6.49 days (*β* = 6.491, *p* < 0.05), column (2) shows an increase of 5.56 days for middle-aged households (*β* = 5.563, *p* < 0.05), while column (3) shows that the impact on older adult households is not significant (*β* = 2.404, *p* > 0.1). Wald tests show that inter-group differences in overall non-farm labor time are significant at the 5% level (*F* = 2.87, *p* = 0.037). Regarding employment location, column (5) shows that obtaining assistance increases local non-farm labor time for middle-aged households by 4.98 days (*β* = 4.983, *p* < 0.05). These results validate hypothesis H4a, indicating that the promoting effect of MFA on non-farm labor time is most significant in prime-age-dominated households with greater labor endowments.

Panel B reports the estimation results for MFA intensity. Regarding overall non-farm labor time, column (1) shows that the coefficient for the logarithm of the assistance amount in youth households is marginally significant at the 10% level (*β* = 0.972, *p* < 0.1), while the coefficients for middle-aged and older adult households do not pass the significance test. For local and non-local non-farm labor time, the coefficients for the logarithm of the assistance amount are insignificant for all household types. Wald tests indicate that inter-group differences in the assistance amount coefficients are not significant for any of the dependent variables (*p* > 0.1). This finding once again confirms the universal judgment of hypothesis H2c regarding the threshold effect.

Combining the findings from Tables 13, 14, the MFA policy exhibits differentiated impact patterns across households at different life cycle stages. On the participation rate dimension, the policy effect primarily benefits older adult households with heavier caregiving burdens, validating H3a. On the labor time dimension, the policy effect primarily benefits prime-age-dominated households with greater labor endowments, validating H4a. This differentiation reveals the deeper structure of the MFA policy effect. Assistance eligibility first helps burdened households take the initial step, transitioning from non-work to work participation decisions. For households already participating in labor, assistance eligibility unlocks their potential for increased labor time input, with this effect being more significant in households with greater labor endowments.

#### 5.4.2 Heterogeneity based on disease type

To examine whether the effect of the MFA policy varies with the severity of health shocks, this study groups households based on disease diagnosis information from medical insurance reimbursement records. The major disease group includes 30 major diseases specified in the Diseases for Special Treatment of Major Illnesses among the Poor Population issued by the National Health Commission, such as malignant tumors, end-stage renal disease, and severe cardiovascular and cerebrovascular diseases, which involve high treatment costs and significant impairment of labor capacity. The chronic disease group encompasses four types of chronic diseases requiring long-term standardized management as specified in the National Basic Public Health Service Standards (Third Edition), including hypertension, type 2 diabetes, tuberculosis, and severe mental disorders. The minor disease group consists of other common and frequently occurring diseases that are short-term and curable. This study employs two-way fixed effects models for group-specific estimations to control for unobserved individual heterogeneity and common time trends.

(1) *Heterogeneous impact on non-farm labor participation rate*. Table 15 reports the estimation results of the impact of MFA on the non-farm labor participation rate of households with different disease types. Panel A uses MFA receipt as the core explanatory variable to test the heterogeneity of the policy breadth effect, corresponding to hypothesis H3b. Panel B uses the logarithm of the MFA amount as the core explanatory variable to test the heterogeneity of the policy intensity effect, corresponding to hypothesis H3d.

**Table 15:** Heterogeneity analysis by health shock type: impact of MFA on NFPR.

Variable	Overall NFPR			Local NFPR			Non-local NFPR		
	Minor	Chronic	Major	Minor	Chronic	Major	Minor	Chronic	Major
	(1)	(2)	(3)	(4)	(5)	(6)	(7)	(8)	(9)
Panel A	MFA breadth								
MFA receipt	0.015	0.017	0.095*	0.015	0.011	0.119**	0.000	0.004	−0.019
	(0.010)	(0.015)	(0.051)	(0.011)	(0.015)	(0.053)	(0.006)	(0.003)	(0.016)
Constant	3.543***	1.923	−0.022	2.937**	1.525	−3.214	0.820*	0.364	2.612***
	(1.308)	(1.624)	(3.593)	(1.234)	(1.562)	(3.597)	(0.452)	(0.258)	(0.901)
Wald test		2.85** (0.038)			2.35* (0.073)			0.26 (0.851)	
Panel B	MFA intensity								
MFA amount (log)	0.002 (0.002)	0.002 (0.002)	0.004 (0.006)	0.001 (0.002)	0.001 (0.002)	0.008 (0.007)	0.001 (0.001)	0.001 (0.000)	−0.003 (0.003)
Constant	3.548*** (1.309)	1.948 (1.625)	−0.512 (3.591)	2.939** (1.235)	1.541 (1.561)	−3.776 (3.615)	0.824* (0.453)	0.369 (0.258)	2.668*** (0.916)
Wald test		1.59 (0.192)			1.03 (0.379)			0.9 (0.444)	
Controls	YES	YES	YES	YES	YES	YES	YES	YES	YES
Household FE	YES	YES	YES	YES	YES	YES	YES	YES	YES
Year FE	YES	YES	YES	YES	YES	YES	YES	YES	YES
*R* ^2^	0.078	0.098	0.097	0.068	0.098	0.096	0.013	0.016	0.034
*N*	10,581	12,007	3,605	10,581	12,007	3,605	10,581	12,007	3,605

MFA denotes Medical Financial Assistance. NFLT denotes non-farm labor time measured in days. FE denotes fixed effects. Youth, middle-aged, and older adult households are classified based on the highest proportion of members aged <18, 18–64, and ≥65, respectively. Wald tests examine inter-group differences in coefficients. Clustered robust standard errors are in parentheses. *, **, and *** denote significance at the 10, 5, and 1% levels, respectively.

From the estimation results in Panel A, obtaining MFA eligibility only has a significant impact on the non-farm labor participation rate of households with major diseases. Regarding the overall non-farm labor participation rate, column (3) shows that the estimated coefficient for major disease households is 0.095, significant at the 10% level. The coefficients for minor disease households in column (1) and chronic disease households in column (2) are not significant. Regarding employment location, this effect is entirely concentrated in the local market. Column (6) shows that the local non-farm labor participation rate for major disease households increases by 11.9 percentage points (*β* = 0.119, *p* < 0.05), while column (9) shows no significant change in the non-local non-farm labor participation rate. Wald tests indicate that inter-group differences in the overall non-farm labor participation rate are significant at the 5% level (*F* = 2.85, *p* = 0.038), and inter-group differences in the local non-farm labor participation rate are marginally significant at the 10% level (*F* = 2.35, *p* = 0.073). These results validate hypothesis H3b, indicating that the promoting effect of MFA on the non-farm labor participation rate is most significant in households with major diseases experiencing the most severe health shocks.

Panel B reports the estimation results for MFA intensity. Regarding the overall non-farm labor participation rate, columns (1) to (3) show that the coefficients for the logarithm of the assistance amount are not significant for any of the three household types. For local and non-local non-farm labor participation rates, the coefficients for the logarithm of the assistance amount are also insignificant for all household types. Wald tests indicate that inter-group differences in the coefficients for assistance amounts are not significant for any of the dependent variables (*p* > 0.1). This result differs from the expectation of hypothesis H3d, as no significant positive impact of the assistance amount on the participation rate is found in households with major diseases.

(2) *Heterogeneous impact on non-farm labor time*. Table 16 reports the estimation results of the impact of MFA on non-farm labor time for households with different disease types. Panel A tests the heterogeneity of the policy breadth effect, corresponding to hypothesis H4b. Panel B tests the heterogeneity of the policy intensity effect, corresponding to hypothesis H4d.

**Table 16:** Heterogeneity analysis by health shock type: impact of MFA on NFLT.

Variable	Overall NFLT			Local NFLT			Non-local NFLT		
	Minor	Chroni	Major	Minor	Chronic	Major	Minor	Chronic	Major
	(1)	(2)	(3)	(4)	(5)	(6)	(7)	(8)	(9)
Panel A	MFA breadth								
MFA receipt	3.789 (3.410)	1.429 (2.978)	36.001*** (8.329)	3.574 (3.318)	2.154 (2.973)	35.836*** (9.231)	−0.215 (1.616)	−0.504 (1.021)	−1.604 (5.374)
Constant	96.215 (266.949)	−860.95*** (319.925)	−2,306.93*** (786.327)	108.867 (260.367)	−791.682** (316.613)	−2,508.80*** (786.502)	−20.781 (91.960)	−41.572 (72.763)	113.708 (238.372)
Wald test		2.76** (0.043)			2.90** (0.035)			0.55 (0.648)	
Panel B	MFA intensity								
MFA amount (log)	0.379 (0.613)	−0.206 (0.462)	3.738*** (1.138)	0.234 (0.608)	−0.092 (0.455)	3.644*** (1.248)	0.087 (0.307)	−0.082 (0.162)	−0.209 (0.809)
Constant	96.87 (266.81)	−858.99*** (320.01)	−2,450.20*** (801.66)	108.85 (260.05)	−788.66** (316.41)	−2,652.97*** (805.94)	−20.26 (92.04)	−42.32 (72.73)	119.23 (239.44)
Wald test		0.53 (0.665)			0.68 (0.566)			0.45 (0.720)	
Controls		YES			YES			YES	
Household FE		YES			YES			YES	
Year FE		YES			YES			YES	
*R* ^2^	0.231	0.223	0.260	0.185	0.203	0.235	0.014	0.015	0.011
*N*	10,581	12,007	3,605	10,581	12,007	3,605	10,581	12,007	3,605

MFA denotes Medical Financial Assistance. NFLT denotes non-farm labor time measured in days. FE denotes fixed effects. Minor, chronic, and major disease groups are classified based on medical insurance reimbursement records. Wald tests examine inter-group differences in coefficients. Clustered robust standard errors are in parentheses. *, **, and *** denote significance at the 10, 5, and 1% levels, respectively.

Panel A shows that the impact pattern of obtaining MFA eligibility on labor time is highly consistent with that on the participation rate. Regarding overall non-farm labor time, column (3) shows that labor time significantly increases by 36.001 days for households with major diseases (*β* = 36.001, *p* < 0.01), while the coefficients for minor disease households in column (1) and chronic disease households in column (2) are not significant. Regarding employment location, this increase is entirely driven by local employment. Column (6) shows that local non-farm labor time for households with major diseases increases by 35.836 days (*β* = 35.836,*p* < 0.01), while column (9) shows no significant change in non-local non-farm labor time. Wald tests indicate that inter-group differences in both overall and local non-farm labor time are significant at the 5% level, with *p*-values of 0.043 and 0.035, respectively. These results validate hypothesis H4b, indicating that the promoting effect of MFA on non-farm labor time is most significant in households with major diseases experiencing the most severe health shocks.

Panel B reports the estimation results for MFA intensity. Regarding overall non-farm labor time, column (3) shows that the coefficient for the logarithm of the assistance amount in major disease households is 3.738, significant at the 1% level, indicating that a one-unit increase in the logarithm of the assistance amount increases non-farm labor time for major disease households by 3.738 days. The coefficients for minor disease households in column (1) and chronic disease households in column (2) are not significant. Regarding employment location, this effect is similarly concentrated in the local market. Column (6) shows that the coefficient for the logarithm of the assistance amount in major disease households for local non-farm labor time is 3.644, significant at the 1% level. It should be noted that Wald tests indicate that inter-group differences in the assistance amount coefficients are not significant, with all p-values greater than 0.1. This means that although the effect is significant within major disease households, it cannot be statistically confirmed that this effect is significantly larger than that in other household types. Compared to the 36-day increase triggered by obtaining eligibility, the approximately 3.7-day increment associated with a one-unit increase in the logarithm of the assistance amount is relatively limited in economic significance.

#### 5.4.3 Heterogeneity based on employment assistance status

To examine whether the effect of the MFA policy varies with the external employment support received by households, this study divides the total sample into two groups: those with employment assistance and those without, based on whether low-income rural households have received government-provided employment support measures such as skills training, labor export services, or public welfare job placement.

(1) *Heterogeneous impact on non-farm labor participation rate*. Table 17 reports the estimation results of the impact of MFA on the non-farm labor participation rate of households with different employment assistance statuses. Panel A uses MFA receipt as the core explanatory variable to test the heterogeneity of the policy breadth effect, corresponding to hypothesis H3c. Panel B uses the logarithm of the MFA amount as the core explanatory variable to test the heterogeneity of the policy intensity effect, corresponding to hypothesis H3d.

**Table 17:** Heterogeneity analysis by employment assistance status: impact of MFA on NFPR.

Variable	Overall NFPR		Local NFPR		Non-local NFPR	
	With EA	Without EA	With EA	Without EA	With EA	Without EA
	(1)	(2)	(3)	(4)	(5)	(6)
Panel A	MFA breadth					
MFA receipt	0.004 (0.006)	0.025** (0.011)	0.005 (0.007)	0.023** (0.011)	−0.001 (0.004)	0.002 (0.003)
Constant	1.245*** (0.299)	−2.910** (1.123)	1.367*** (0.337)	−3.045*** (1.099)	−0.151 (0.186)	0.248 (0.167)
Wald test	0.28 (0.600)		2.77* (0.097)		8.45*** (0.004)	
Panel B	MFA intensity					
MFA amount (log)	−0.000 (0.001)	0.004** (0.002)	0.000 (0.001)	0.004** (0.002)	−0.000 (0.001)	0.001 (0.000)
Constant	1.247*** (0.300)	−2.855** (1.123)	1.372*** (0.336)	−3.001*** (1.099)	−0.154 (0.186)	0.256 (0.168)
Wald test	0.00 (0.994)		1.04 (0.308)		6.26** (0.013)	
Controls	YES	YES	YES	YES	YES	YES
Household FE	YES	YES	YES	YES	YES	YES
Year FE	YES	YES	YES	YES	YES	YES
*R* ^2^	0.131	0.117	0.144	0.106	0.020	0.010
*N*	12,173	14,020	12,173	14,020	12,173	14,020

MFA denotes Medical Financial Assistance. NFPR denotes non-farm labor participation rate. EA denotes employment assistance. FE denotes fixed effects. Households are classified based on whether they have received government-provided employment support measures. Wald tests examine inter-group differences in coefficients. Clustered robust standard errors are reported in parentheses. *, **, and *** denote significance at the 10, 5, and 1% levels, respectively.

From the estimation results in Panel A, obtaining MFA eligibility has a significant positive impact on the non-farm labor participation rate of households without employment assistance, while having no significant impact on households with employment assistance. Regarding the overall non-farm labor participation rate, column (2) shows that the estimated coefficient for households without employment assistance is 0.025, significant at the 5% level. Column (1) shows that the coefficient for households with employment assistance is 0.004 and is not significant. Regarding employment location, column (4) shows that the local non-farm labor participation rate for households without employment assistance increases by 2.3 percentage points (*β* = 0.023, *p* < 0.05), while column (3) shows no significant change for households with employment assistance. Wald tests indicate that inter-group differences in the local non-farm labor participation rate are marginally significant at the 10% level (*F* = 2.77, *p* = 0.097), and inter-group differences in the non-local non-farm labor participation rate are significant at the 1% level (*F* = 8.45, *p* = 0.004). These results validate hypothesis H3c, indicating that the promoting effect of MFA on the non-farm labor participation rate is most significant in households without employment assistance that lack external employment support.

Panel B reports the estimation results for MFA intensity. Regarding the overall non-farm labor participation rate, column (2) shows that the coefficient for the logarithm of the assistance amount in households without employment assistance is 0.004, significant at the 5% level. The coefficient for households with employment assistance in column (1) is not significant. For the local non-farm labor participation rate, column (4) shows that the coefficient for the logarithm of the assistance amount in households without employment assistance is also 0.004, significant at the 5% level. Wald tests indicate that inter-group differences in the non-local non-farm labor participation rate are significant at the 5% level (*F* = 6.26, *p* = 0.013). These results validate hypothesis H3d, indicating that in households without employment assistance, the assistance amount has a significant positive impact on the non-farm labor participation rate.

(2) *Heterogeneous impact on non-farm labor time*. Table 18 reports the estimation results of the impact of MFA on non-farm labor time for households with different employment assistance statuses. Panel A tests the heterogeneity of the policy breadth effect, corresponding to hypothesis H4c. Panel B tests the heterogeneity of the policy intensity effect, corresponding to hypothesis H4d.

**Table 18:** Heterogeneity analysis by employment assistance status: impact of MFA on NFLT.

Variable	Overall NFLT		Local NFLT		Non-local NFLT	
	With EA	Without EA	With EA	Without EA	With EA	Without EA
	(1)	(2)	(3)	(4)	(5)	(6)
Panel A	MFA breadth					
MFA receipt	4.549** (1.791)	0.920 (2.278)	4.621** (2.024)	0.213 (2.139)	−0.712 (1.409)	1.321 (0.963)
Constant	−523.316*** (125.172)	−466.597*** (174.840)	−347.511*** (129.906)	−450.290*** (154.997)	−155.142* (85.670)	−7.868 (56.271)
Wald test	0.68 (0.412)		0.02 (0.901)		0.32 (0.573)	
Panel B	MFA intensity					
MFA amount (log)	0.438 (0.280)	0.284 (0.305)	0.505 (0.324)	0.145 (0.285)	−0.161 (0.235)	0.229 (0.143)
Constant	−518.887*** (125.227)	−462.799*** (175.643)	−342.676*** (129.338)	−448.280*** (155.903)	−156.359* (85.763)	−4.954 (56.091)
Wald test	0.85 (0.356)		0.23 (0.634)		0.01 (0.906)	
Controls	YES	YES	YES	YES	YES	YES
Household FE	YES	YES	YES	YES	YES	YES
Year FE	YES	YES	YES	YES	YES	YES
*R* ^2^	0.128	0.124	0.070	0.097	0.043	0.024
*N*	12,173	14,020	12,173	14,020	12,173	14,020

MFA denotes Medical Financial Assistance. NFLT denotes non-farm labor time measured in days. EA denotes employment assistance. FE denotes fixed effects. Households are classified based on whether they have received government-provided employment support measures. Wald tests examine inter-group differences in coefficients. Clustered robust standard errors are in parentheses. *, **, and *** denote significance at the 10, 5, and 1% levels, respectively.

Panel A shows that obtaining MFA eligibility has a significant positive impact on the non-farm labor time of households with employment assistance, while having no significant impact on households without employment assistance. Regarding overall non-farm labor time, column (1) shows that labor time for households with employment assistance increases by 4.55 days (*β* = 4.549, *p* < 0.05), while column (2) shows that the coefficient for households without employment assistance is not significant. Regarding employment location, column (3) shows that local non-farm labor time for households with employment assistance increases by 4.62 days (*β* = 4.621, *p* < 0.05). Wald tests indicate that inter-group differences are not significant for any of the dependent variables (*p* > 0.1). These results are consistent with the expectation of hypothesis H4c, indicating that the promoting effect of MFA on non-farm labor time is more significant in households with employment assistance. However, it should be noted that the inter-group differences do not reach statistical significance, meaning that although the point estimates suggest a larger effect for households with employment assistance, this difference cannot be reliably detected statistically.

Panel B reports the estimation results for MFA intensity. For overall non-farm labor time, local non-farm labor time, and non-local non-farm labor time, the coefficients for the logarithm of the assistance amount are not significant for either households with employment assistance or those without. Wald tests indicate that inter-group differences in the assistance amount coefficients are not significant for any of the dependent variables (*p* > 0.1). These results do not support hypothesis H4d in the employment assistance dimension.

## 6 Discussion

### 6.1 Discussion of baseline effects

Obtaining MFA eligibility significantly increases the non-farm labor participation rate and non-farm labor time of low-income rural households, with this effect primarily concentrated in the local non-farm employment market. This finding contributes meaningfully to existing research.

This study provides new evidence for the empowering effect of medical security from the perspective of labor supply. Some studies have revealed that medical security may suppress labor supply through income effects (23) or affect labor mobility through employment lock effects (18). However, this study finds that MFA generates a significant positive incentive for the labor supply of low-income rural households. This result echoes Strumpf’s (16) findings on the introduction of Medicaid in the United States. Strumpf (16) noted that the positive effects of public health insurance through health improvement may offset or even exceed its potential labor disincentive effects. In the specific institutional context of China’s rural MFA, this study provides direct evidence for the existence of the health empowerment pathway.

In addition to the overall promoting effect, the locational structure of the policy effect is noteworthy. The results indicate that obtaining MFA eligibility primarily promotes local non-farm labor participation and increases local non-farm labor time, while having a very limited impact on non-local employment. This finding complements the conclusions of Shen et al. (13) regarding the NCMS promoting non-farm labor participation. Shen et al. (13) revealed the overall promoting effect of medical insurance on labor supply, while this study further reveals the locational structure of this effect, demonstrating that it is primarily manifested as stimulating local employment rather than promoting non-local mobility. This finding confirms the core assertion of Doeringer and Piore’s (52) internal labor market theory that workers entering non-local markets face entry barriers constituted by information asymmetry and social network deficits.

Further decomposition of the policy effect reveals significant differences between the eligibility effect and the intensity effect of MFA. Obtaining MFA eligibility is the critical factor triggering household labor supply responses, while the marginal incentive effect of increased assistance amounts on labor supply is very weak. This finding can be explained by the threshold characteristic of health capital investment. Grossman’s health capital theory implies an important premise that health investment has a minimum effective scale. Only when investment reaches a certain threshold can substantial restoration of health capital stock be achieved (1). Obtaining MFA eligibility itself enables households to cross this investment threshold, initiating the health restoration process. Once the assistance amount reaches a level sufficient to cover basic medical needs, the marginal restorative effect of additional amounts on health capital may diminish to insignificance. Research by Van Zon and Muysken (71) on health and endogenous growth similarly points out that the promoting effect of health investment on labor productivity exhibits stage-specific characteristics.

Precautionary savings theory suggests that expenditure uncertainty caused by health shocks forces households to strengthen precautionary savings (70). Obtaining MFA eligibility, by stabilizing household expectations about future medical expenditures, may be more effective in alleviating this budget constraint effect than increasing small amounts of assistance. Existing research has primarily focused on the binary effect of having or not having medical insurance, with insufficient attention to the marginal effects of policy support intensity. This finding echoes the conclusions of Gooptu et al. (19) on Medicaid expansion, which found that the employment status of low-income adults did not change significantly in the 15 months following expansion, suggesting that obtaining eligibility itself may constitute a critical factor for policy effects. Building on this, this study further extends this threshold effect from the participation rate dimension to the labor time dimension and systematically examines its manifestation across different employment locations.

### 6.2 Discussion of heterogeneous effects

This study examines the heterogeneity of MFA policy effects across three dimensions: family life cycle, disease type, and employment assistance status. The results reveal systematic differentiation patterns across different groups.

(1) As hypothesized (H3a and H4a), in the family life cycle dimension, the effect of obtaining MFA eligibility on the non-farm labor participation rate is most significant in older adult households, while its effect on non-farm labor time is most significant in prime-age households. This differentiation aligns with the core expectations of Becker’s and Gronau’s household production theories (2, 3). Older adult households face greater health risks and caregiving burdens, with health shocks imposing more pronounced constraints on their participation decisions. Therefore, MFA primarily helps these households cross the participation threshold. In contrast, prime-age households have more abundant labor endowments and can more flexibly extend labor time following health improvements, making the policy effect more pronounced in the labor time dimension. This finding supports Liu’s argument that the effects of medical security vary across different stages of the life cycle (29).
(2) Consistent with H3b and H4b, in the disease type dimension, the effect of obtaining MFA eligibility on non-farm labor supply is most significant in households with major diseases. The response intensity of major disease households is much higher than that of minor disease and chronic disease households in both the participation rate and labor time dimensions. This finding is strongly aligned with the core logic of Grossman’s health capital theory (1). Major disease households experience the most severe health shocks and the greatest depreciation of health capital, so the marginal benefits of MFA through the restoration of health human capital are naturally the highest. Kim’s research using Korean data found that the main channel through which health shocks lead to poverty after 2 years is declining labor capacity rather than medical expenses themselves (11). This study’s findings echo Kim’s from the perspective of policy intervention. Since declining labor capacity is a key pathway through which health shocks cause poverty, MFA policies targeting the restoration of health capital should yield the most significant responses in major disease households.

In addition to the significant effect of eligibility attainment, the marginal impact of assistance amounts is also noteworthy. The non-farm labor time of major disease households responds significantly to assistance amounts, but inter-group differences do not reach statistical significance. This result has two implications. On one hand, the sensitivity of major disease households to assistance amounts aligns with the expectation of hypothesis H4d. This can be explained by the theoretical analysis of Olivella and Vera-Hernandez (50) on how disease severity affects household economic shocks. Major disease households face persistent treatment cost pressures, and additional financial support helps maintain treatment continuity, thereby contributing marginally to the restoration of labor capacity. On the other hand, the insignificance of inter-group differences and the limited magnitude of effects further reinforce this study’s core judgment regarding the threshold effect. Compared to the 36-day increase triggered by obtaining eligibility, the approximately 3.7-day increment associated with a one-unit increase in the logarithm of the assistance amount is relatively limited in economic significance. This pattern is highly consistent with the characteristics observed in the baseline regression of eligibility dominance and weak intensity effects, indicating that even among the major disease group most in need of sustained financial support, the marginal effect of policy incentives is still constrained by the threshold characteristic of health investment.

(3) As predicted by H3c and H4c, in the employment assistance dimension, the promoting effect of obtaining MFA eligibility on the non-farm labor participation rate is most significant in households without employment assistance, while the promoting effect on non-farm labor time is more significant in households with employment assistance. This differentiation reveals the complementary relationship between MFA and employment assistance policies. For households lacking external employment support, their labor force is positioned at the market periphery, facing higher information barriers and entry costs. MFA, through health restoration and time release, first helps these households cross the participation threshold, facilitating their transition from market periphery to market entry. For households already receiving employment assistance, they possess some foundation of market connection and reserves of employment capacity. The role of MFA for these households is more reflected in extending their effective labor time. This finding echoes the research of Zhang et al. (57) on the promoting effect of poverty alleviation policies on labor supply in local non-migrant households.

Similar to the disease type dimension, the effect of assistance amounts in the employment assistance dimension also exhibits meaningful characteristics. The non-farm labor participation rate of households without employment assistance responds significantly to assistance amounts, validating hypothesis H3d that the most vulnerable groups are more sensitive to changes in assistance amounts. This finding is logically consistent with García-Gómez et al.’s (35) conclusion that health shocks have more severe effects on vulnerable groups. The more vulnerable the group, the more sensitive their marginal response to policy support. However, it should be noted that even in households without employment assistance, the magnitude of the impact of assistance amounts on labor participation rates is much smaller than the effect of achieving eligibility, once again confirming the universality of the threshold effect.

### 6.3 Comprehensive discussion

Synthesizing the above analysis, this study reveals the fundamental picture of the impact of the MFA policy on the non-farm labor supply of low-income rural households. Overall, obtaining MFA eligibility significantly stimulates households’ non-farm labor supply, with this effect exhibiting a clear localization bias and a distinct threshold characteristic. At the heterogeneity level, the policy effect shows systematic differentiation across different groups. The participation rate dimension primarily benefits older adult households with heavier burdens, major disease households, and households without employment assistance. The labor time dimension primarily benefits prime-age households with more abundant endowments, major disease households, and households with employment assistance.

It is worth emphasizing that the basic judgment of eligibility effect dominance and weak intensity effect is consistently confirmed in both the baseline regression and heterogeneity analysis. Even among major disease households most in need of sustained financial support and the most vulnerable households without employment assistance, the incentive effect of achieving eligibility far exceeds the marginal contribution of increased assistance amounts. This pattern reveals the core mechanism through which the MFA policy incentivizes labor supply. It lies not in the accumulation of assistance amounts, but rather in enabling more vulnerable households to cross the eligibility threshold, thereby triggering the chain reaction of health capital restoration and family time release.

In their Lancet review, Jan et al. pointed out that financial risk protection for non-communicable diseases requires comprehensive policy frameworks (8). The findings of this study provide micro-level evidence from low-income groups for this framework, revealing the unique positioning of the MFA policy within a comprehensive framework. It is not only a tool for risk protection but also an important institutional arrangement for stimulating household development capacity.

### 6.4 Indirect evidence for the proposed mechanisms

The theoretical framework proposed two pathways through which MFA may affect non-farm labor supply: health capital restoration and household time release. The heterogeneity results provide suggestive evidence consistent with these mechanisms.

(1) The health capital restoration pathway predicts that households with greater potential for health improvement should exhibit stronger labor supply responses. Two patterns support this prediction. First, as shown in Table 16, households with major diseases—who experience the most severe health impairments—exhibit a 36.00-day increase in non-farm labor time after receiving MFA, which is more than ten times the effect observed for households with minor or chronic diseases (both statistically insignificant). Second, Table 13 shows that the increase in the non-farm labor participation rate is largest for older adult households (3.6 percentage points), who typically suffer from greater health depreciation and thus have more room for health improvement. Both patterns align with the health capital restoration pathway.
The time release pathway predicts that households with heavier caregiving responsibilities should benefit more from MFA, as it can free up time previously devoted to patient care. The results in Table 14 show that middle-aged households, who typically bear the dual burden of caring for both older adult parents and children, experience a significant increase in non-farm labor time of 5.56 days (*p* < 0.05). In contrast, older adult households, whose caregiving responsibilities primarily involve receiving care rather than providing it, show no significant increase in labor time (2.40 days, *p* > 0.1). This contrast supports the time release mechanism.
The localization bias documented in the baseline results (93.6% of the increase in labor time occurs locally) is also consistent with both mechanisms. Released labor time is more likely to be allocated to local employment due to health thresholds, migration costs, and family responsibilities, as argued in the theoretical framework.

Taken together, the observed heterogeneity patterns are fully consistent with the predictions of the proposed mechanisms, providing strong supportive evidence for the pathways through which MFA affects non-farm labor supply.

## 7 Conclusion and future research prospects

### 7.1 Conclusion

Using two-wave household panel data from 2021 to 2022 in County L, a nationally designated deep poverty-stricken county in the Yanshan-Taihang Mountain region of Hebei Province, this study systematically examines the impact of the Medical Financial Assistance policy on the non-farm labor supply of low-income rural households by employing two-way fixed effects models. The main research conclusions are as follows.

First, obtaining MFA eligibility has a significant positive effect on the non-farm labor supply of low-income rural households. After controlling for household heterogeneity and time trends, obtaining MFA increases the household’s overall non-farm labor participation rate by 2.0 percentage points and overall non-farm labor time by 4.87 days. This effect primarily stems from eligibility attainment itself rather than increases in assistance amounts. A 1 % increase in the assistance amount only increases the non-farm labor participation rate by 0.002 percentage points, and its impact on non-farm labor time is not significant. This indicates that the labor supply incentive effect of the MFA policy exhibits a clear threshold characteristic. Eligibility attainment is the critical factor triggering household behavioral responses, while the marginal incentive effect of increased amounts is very weak.

Second, the policy effect of MFA exhibits a significant localization bias. Obtaining MFA eligibility increases a household’s local non-farm labor participation rate by 1.7 percentage points and local non-farm labor time by 4.56 days, accounting for 93.6 percent of the total increase. In contrast, the impacts on non-local non-farm labor participation rates and labor time are not significant. This finding confirms the core assertion of internal labor market theory that workers entering non-local markets face entry barriers constituted by multiple factors, including information asymmetry, social network deficits, health thresholds, and family caregiving responsibilities. Released labor is preferentially allocated to the local employment market, forming the locational structure characteristic of the policy effect.

Third, the effect of the MFA policy exhibits systematic heterogeneity across different groups. In terms of participation rates, the policy effect primarily benefits older adult households with heavier caregiving burdens, major disease households experiencing the most severe health shocks, and households without employment assistance that lack external support. In terms of labor time, the policy effect primarily benefits prime-age households with more abundant labor endowments, major disease households, and households with employment assistance. This differentiation reveals the logic of the MFA policy’s mechanism. Assistance eligibility first helps the most burdened households cross the participation threshold, facilitating their transition from non-work to work. For households already participating in labor, assistance eligibility further enhances their potential for increased labor time input.

Fourth, the marginal effect of MFA manifests only in specific groups and has relatively limited economic significance. The non-farm labor time of major disease households responds significantly to assistance amounts, but inter-group differences do not reach statistical significance. The non-farm labor participation rate of households without employment assistance responds significantly to assistance amounts, but the magnitude of the impact is much smaller than the effect of eligibility attainment. This finding further reinforces the core judgment of eligibility effect dominance and weak intensity effect. It indicates that even among major disease households with the greatest need for sustained financial support and the most vulnerable households without employment assistance, the marginal effect of policy incentives is still constrained by the threshold characteristic of health investment.

It should be noted that the conclusions of this study are derived from household survey data from County L, a nationally designated deep poverty-stricken county in the Yanshan-Taihang Mountain region of Hebei Province. As a typical deeply impoverished region, this area has specific regional characteristics in terms of economic and social development levels, medical security policy implementation, and labor market development. Therefore, the extrapolation of this study’s conclusions requires consideration of regional differences and further validation in different institutional environments and stages of economic development. However, the policy effect pattern of eligibility dominance and weak intensity, along with the basic logic of localization bias and heterogeneous differentiation revealed by this study, provides certain theoretical reference value for understanding the developmental function of MFA policies among low-income groups.

### 7.2 Implications

#### 7.2.1 Theoretical implications

This study extends the policy effect of MFA from ex-post compensation to ex-ante risk smoothing, and from income transfer to labor supply incentives, revealing multiple functions of the social security system.

Precautionary savings theory emphasizes the inhibitory effect of uncertainty expectations on household resource allocation, and subsequent research further confirms that expenditure uncertainty caused by health shocks forces households to strengthen their precautionary savings (75). This study applies this theoretical framework to the MFA context and finds that obtaining MFA eligibility, by stabilizing household expectations about future medical expenditures, alleviates the crowding out of liquidity by precautionary savings. This finding indicates that MFA not only has an ex-post compensation function but also plays an ex-ante risk smoothing role by reducing expenditure uncertainty, thereby extending the policy connotation of MFA from mere ex-post compensation to ex-ante risk smoothing.

Further decomposition of the policy effect reveals that obtaining MFA eligibility is the critical factor triggering household labor supply responses, while the marginal incentive effect of increased assistance amounts on labor supply is minimal. This finding aligns with the implicit premise in Grossman’s health capital theory that health investment has a minimum effective scale (1). Health investment may need to reach a certain threshold to achieve a substantial restoration of health capital stock. Attaining eligibility itself enables households to cross this threshold, initiating the health restoration process. Thus, this study extends the analytical perspective of health capital theory from the quantity of health investment to the threshold of health investment, suggesting that when examining the impact of medical security on labor supply, attention should be paid to the threshold characteristics of policy intervention.

The policy effect of MFA is not homogeneous but exhibits systematic differentiation across three dimensions: family life cycle, disease type, and employment assistance status. The effect on the participation rate primarily benefits older adult households, households with major diseases, and those without employment assistance. The effect on labor time primarily benefits prime-age households, households with major diseases, and those with employment assistance. The promoting effect of MFA on the participation rate of older adult households is particularly significant. This finding provides new empirical support for household production theory (2, 3), confirming that health shocks may suppress overall labor supply through intra-household caregiving demands, and that MFA, by restoring patient health and alleviating caregiving burdens, releases locked household labor. These characteristics of heterogeneity collectively indicate that the effectiveness of the policy depends on specific household characteristics and shock conditions. This promotes a paradigm shift in MFA policy evaluation from merely assessing whether the policy is effective on average to deeply exploring for whom it is effective and under what conditions, providing a theoretical foundation for constructing a precise policy analysis framework.

#### 7.2.2 Practical implications

The findings of this study have the following reference value for the optimization design of MFA policies. It should be noted that the following recommendations are based on an empirical analysis of a nationally designated deep poverty-stricken county in the Yanshan-Taihang Mountain region of Hebei Province, China. Regions differ in economic development levels, medical security policy implementation, and labor market development, so the application of these findings requires careful adjustment based on local conditions.

First, expanding coverage should be prioritized over increasing assistance standards. This study finds that obtaining MFA eligibility is the critical factor triggering household labor supply responses, while the marginal incentive effect of increased assistance amounts is very weak. This implies that expanding coverage is more policy-efficient than increasing assistance standards. It is recommended that, within fiscal affordability, the criteria for household income assessment be gradually relaxed to include more households vulnerable to poverty. Simultaneously, the application process should be simplified, documentation requirements reduced, and review cycles shortened to lower household application costs, enabling policy benefits to reach target groups in a timely manner.

Second, differentiated interventions should be implemented for different household types. The heterogeneity analysis in this study indicates that the effect of the MFA policy exhibits systematic variation across different groups. For older adult households, households with major diseases, and households without employment assistance, the labor participation rate is more sensitive to eligibility attainment. It is recommended that these groups be prioritized as key targets for policy outreach and application assistance. Village or community workers could proactively visit to inform them about the policy and assist with application materials. For prime-age households and those receiving employment assistance, their labor time is more responsive to eligibility attainment. For such households, after obtaining assistance, employment matching and skills training could be enhanced to help them achieve fuller labor participation.

Third, coordination between MFA and local employment promotion policies should be strengthened. This study finds that the policy effect is primarily concentrated in the local non-farm employment market, indicating that the availability of local employment opportunities directly affects the realization of policy effects. It is recommended that human resources and social security departments establish information-sharing mechanisms with rural revitalization departments. After a household obtains MFA, the system could automatically send information to employment service departments, where employment service specialists proactively contact households, understand their employment needs, and provide job recommendations. For assistance recipients with labor capacity, priority could be given to arranging public welfare positions and providing vocational skills training, enabling released labor to smoothly enter the local employment market, thereby forming a virtuous cycle of health security, labor participation, and income improvement.

Fourth, a full-cycle support chain should be constructed for households affected by major diseases. This study finds that these households exhibit the most significant policy response to MFA, but the marginal effect of assistance amounts is relatively limited. It is recommended that a support system be developed across three stages: in-event emergency response, post-event recovery, and ex-ante prevention. In the in-event emergency response stage, the administrative process should be optimized to ensure timely and complete settlement of assistance funds, resolving households’ payment crises. In the post-event recovery stage, laborers from major disease households during the rehabilitation period should be included in the scope of key employment assistance, with rehabilitation guidance and job matching services provided. This is a crucial step in translating health restoration into labor supply. In the ex-ante prevention stage, coordination with basic public health services should be strengthened, but resource allocation should prioritize the in-event emergency response and post-event recovery stages.

Fifth, a cross-departmental dynamic monitoring and precise assistance mechanism should be established. The effectiveness of MFA requires synergy with basic medical insurance, catastrophic illness insurance, as well as employment assistance and industrial support policies. It is recommended that a cross-departmental information-sharing platform be created to break down data barriers between medical security, health, human resources and social security, and rural revitalization departments, enabling dynamic monitoring of populations vulnerable to falling back into poverty. For households identified through monitoring as potentially facing distress, an early warning mechanism could be triggered promptly, with relevant departments jointly assessing and implementing precise interventions, promoting effective coordination between health security and employment support, industrial support, and other policies, thereby providing institutional safeguards for consolidating and expanding the achievements of poverty alleviation.

Sixth, policy trade-offs and potential tensions should be carefully addressed. The finding that the marginal effect of MFA amounts is very weak raises a cost–benefit question. Specifically, should policy focus shift from raising subsidy standards to expanding the coverage scope? The results suggest that achieving eligibility is the critical trigger for labor supply responses, while increasing amounts yield little additional benefit. Therefore, reallocating limited fiscal resources from increasing per capita assistance to covering more eligible households would likely yield higher aggregate welfare gains. However, this trade-off must be balanced against the need to provide adequate support for major disease households, which still show some sensitivity to assistance amounts. A hybrid approach may be optimal. This approach would maintain basic reimbursement levels for all recipients while using targeted top-ups for major disease cases. Another potential tension arises from the observed localization bias. MFA primarily stimulates local non-farm employment, aligning with the goal of rural revitalization by strengthening local economies. However, overly generous local welfare packages combining MFA with local employment subsidies might inadvertently reduce labor mobility, creating friction with the strategic objective of a unified national labor market. Policymakers should weigh the short-term benefits of local employment against the long-term efficiency gains from labor mobility. One possible resolution is to design MFA benefits to be portable across regions. This would ensure that recipients do not lose assistance when seeking employment outside their home county. Alternatively, local employment support could be structured as temporary transition assistance rather than permanent subsidies. Such an approach would encourage mobility once households recover from health shocks.

### 7.3 Limitations and prospects

This study has several limitations that suggest directions for future inquiry. The two-year panel data and single-county sample, while ensuring rigorous causal identification, constrain the examination of long-term policy dynamics and external validity. The effects of MFA on labor supply may exhibit different trajectories over longer time horizons, and the findings reflect the specific institutional context of a deep poverty county in Hebei Province. Future research could employ longer panel data and multi-region samples to trace the dynamic trajectories of policy effects and examine their robustness across heterogeneous contexts, thereby deepening the understanding of how MFA empowers household development. Furthermore, the proposed mechanisms—health capital restoration and family time release—are supported only indirectly by the heterogeneity patterns observed in this study. Direct mediation tests were not feasible due to the absence of direct measures of health status and caregiving time in the data. Future research should incorporate such measures to formally test these pathways.

## Data Availability

The original contributions presented in the study are included in the article/Supplementary material, further inquiries can be directed to the corresponding authors.
